# Quality control in functional MRI studies with MRIQC and fMRIPrep

**DOI:** 10.3389/fnimg.2022.1073734

**Published:** 2023-01-12

**Authors:** Céline Provins, Eilidh MacNicol, Saren H. Seeley, Patric Hagmann, Oscar Esteban

**Affiliations:** ^1^Department of Radiology, Lausanne University Hospital and University of Lausanne, Lausanne, Switzerland; ^2^Department of Neuroimaging, Institute of Psychiatry, Psychology and Neuroscience, King's College London, London, United Kingdom; ^3^Department of Psychiatry, Icahn School of Medicine at Mount Sinai, New York, NY, United States

**Keywords:** quality control, quality assessment, fMRI, MRIQC, fMRIPrep, exclusion criteria, neuroimaging

## Abstract

The implementation of adequate quality assessment (QA) and quality control (QC) protocols within the magnetic resonance imaging (MRI) research workflow is resource- and time-consuming and even more so is their execution. As a result, QA/QC practices highly vary across laboratories and “MRI schools”, ranging from highly specialized knowledge spots to environments where QA/QC is considered overly onerous and costly despite evidence showing that below-standard data increase the false positive and false negative rates of the final results. Here, we demonstrate a protocol based on the visual assessment of images one-by-one with reports generated by MRIQC and fMRIPrep, for the QC of data in functional (blood-oxygen dependent-level; BOLD) MRI analyses. We particularize the proposed, open-ended scope of application to whole-brain voxel-wise analyses of BOLD to correspondingly enumerate and define the exclusion criteria applied at the QC checkpoints. We apply our protocol on a composite dataset (*n* = 181 subjects) drawn from open fMRI studies, resulting in the exclusion of 97% of the data (176 subjects). This high exclusion rate was expected because subjects were selected to showcase artifacts. We describe the artifacts and defects more commonly found in the dataset that justified exclusion. We moreover release all the materials we generated in this assessment and document all the QC decisions with the expectation of contributing to the standardization of these procedures and engaging in the discussion of QA/QC by the community.

## 1. Introduction

Quality assessment (QA) and quality control (QC) of magnetic resonance imaging (MRI), implemented at several stages of the processing and analysis workflow, are critical for the reliability of the results. QA focuses on ensuring the research workflow produces data of “sufficient quality” (e.g., identifying a structured artifact caused by an environmental condition that can be actioned upon so that it doesn't replicate prospectively in future acquisitions). On the other hand, QC excludes poor-quality data from a dataset so that they do not continue through the research workflow and potentially bias results. Indeed, below-standard MRI data increase the false positive and false negative rates in the final analyses (Power et al., 2012; Alexander-Bloch et al., 2016; Ducharme et al., 2016; Zalesky et al., 2016). For example, Power et al. (2012) showed that unaccounted-for head motion in functional MRI (fMRI) data introduces systematic but spurious spatial correlations that are wrongly interpreted as functional brain connectivity.

Despite efforts toward automation, the implementation of QA/QC checkpoints remains unstandardized and typically involves the screening of the images one by one. Therefore, QA/QC is time-consuming and frequently seen as overly onerous to the development of projects. In the absence of a consensus on systematic approaches to QA/QC and corresponding data curation protocols, laboratories currently rely on their internal know-how. Such knowledge is generally acquired through individual researchers (here, referred to as “raters”) repeatedly screening data. Thus, the knowledge is usually contingent on the context of the studies for which they are acquired and local practices rather than some principled definition of quality criteria that generalize across applications. This leads to a wide variety of QA/QC procedures and protocols across institutions, which add to the inherently large intra- and inter-rater variabilities given a specific QA/QC approach. Therefore, appropriate protocols and tools are required to make QA/QC more consistent across institutions and improve intra- and inter-rater reliability. Substantial work has been proposed to provide efficient interfaces such as MRIQC (Esteban et al., 2017), MindControl (Keshavan et al., 2018) or Swipes4Science (Keshavan et al., 2019). Large consortia have also made remarkable investments in this important task and have developed QA/QC protocols, e.g., the Human Connectome Project (Marcus et al., 2013) or the INDI initiative (QAP; Shehzad et al., 2015). One related but conceptually innovative approach was proposed for the QC of the MRI data of the UK Biobank (Alfaro-Almagro et al., 2018), where quality was defined in a more utilitarian manner as the success of downstream processing. With the rise of large-scale datasets such as the UK Biobank, manually checking the data becomes infeasible. Alfaro-Almagro et al. (2018) described an automated QC approach wherein raw data were screened for having the wrong dimensions, corrupted, missing, or otherwise unusable, and excluded from further preprocessing (first checkpoint). The second checkpoint was applying a supervised learning classifier to the T_1_-weighted (T1w) images. Although image exclusions often occurred in response to qualitative issues on images (e.g., visual identification of artifacts), some images were discarded without straightforward mapping to quality issues, and the classifier was only trained to identify problems in T1w images, so it could not be applied to BOLD data or other modalities. Many researchers have similarly attempted automation, either by relying on no-reference (as no ground truth is available) image quality metrics (IQMs) to train a machine learning model (Mortamet et al., 2009; Shehzad et al., 2015; Esteban et al., 2017) or by training deep models on 3D images directly (Garcia et al., 2022). However, predicting the quality of images acquired at a new site yet unseen by the model remains a challenging problem (Esteban et al., 2017, 2018). Another challenge to developing deep models is the need for large datasets with usable and reliable QA/QC annotations for training. Moreover, the QA/QC annotations must be acquired across sites and rated by many individuals to ensure generalizability (Keshavan et al., 2019).

Here, we demonstrate a protocol for the QC of task-based and resting-state fMRI studies. This contribution is part of the research topic “Demonstrating Quality Control (QC) Procedures in fMRI.” The participants of the research topic were given a composite dataset with anatomical and functional data selected from published studies to demonstrate QC protocols in practice. We describe how the overall application scope (that is, the intended use of the data) determines how QC is carried out and define the exclusion criteria for anatomical (T_1_-weighted; T1w) and functional (blood-oxygen dependent-level; BOLD) images at two QC checkpoints accordingly. We first performed QC of the unprocessed data using the MRIQC visual reports (Esteban et al., 2017). Second, for the data that surpassed this first checkpoint, we assessed the results of minimal preprocessing using the fMRIPrep visual reports (Esteban et al., 2019). Thus, reaching a consensus on the definition of QA/QC evaluation criteria and establishing standard protocols to ascertain such criteria are the keystone toward more objective QA/QC in fMRI research.

## 2. Methods

### 2.1. Data

We used the data collection preselected by the research topic organizers to showcase examples of each exclusion criterion. The dataset gathers resting-state and task fMRI data from several open, public repositories (Biswal et al., 2010; Di Martino et al., 2014; Markiewicz et al., 2021). Therefore, the dataset is eminently multi-site and highly diverse in acquisition devices, parameters, and relevant settings. The selection criteria of datasets and subjects were not disclosed to the research topic participants. The dataset is split into two cohorts: subjects with resting-state scans and subjects with task scans. Every subject has one T1w image and one or two BOLD fMRI scans. Data were released following the Brain Imaging Data Structure (BIDS; Gorgolewski et al., 2016).

### 2.2. Scope of application

Considering the dataset's characteristics, we narrowed the planned analysis's scope to “whole-brain, voxel-wise analyses of spatially standardized task and resting-state BOLD fMRI.” Note that by “whole-brain”, we mean cortex and subcortical structures but not cerebellum because we expected those regions to fall outside of the field of view in a number of the BOLD datasets. For the implementation of such an application, we propose our fMRI protocol (Esteban et al., 2020), which uses fMRIPrep to prepare the data for analysis. fMRIPrep was executed with default settings (for the exact description of the preprocessing see Supplementary material, section 4). Therefore, data are spatially standardized into the MNI152NLin2009cAsym space (Fonov et al., 2009) accessed with TemplateFlow (Ciric et al., 2022). The protocol involves an initial QC checkpoint implemented with MRIQC and a second QC checkpoint on the outputs of fMRIPrep.

### 2.3. QC protocol

#### 2.3.1. Standard operating procedures (SOPs)

To formalize the scope and the QA/QC criteria and protocols, we proposed our MRIQC-SOPs template (https://github.com/nipreps/mriqc-sops) as a scaffold to create custom standard operating procedures (SOPs) documents tailored to the specific project and maintained under version control. We demonstrated MRIQC-SOPs to create the corresponding documentation of this study. These SOPs contain the lists presented in Tables 1–3 and the QC criteria details laid out in Section 2.4 in a format adapted to the SOPs. The SOPs documents can be visualized at http://www.axonlab.org/frontiers-qc-sops/ and can be accessed as stated in the Data and Software availability statement.

**Table 1:** Resting-state and task fMRI exclusion criteria based on the MRIQC visual report.

QC of unprocessed fMRI data based on MRIQC visual report	A) Artifactual structures in the background
	B) Susceptibility distortion artifacts
	BA) Signal drop-out
	BB) Brain distortions
	C) Aliasing ghosts
	D) Wrap-around that overlaps with the brain
	E) Structured crown region in the carpet plot
	EA) due to motion peaks
	EB) due to periodic motion
	EC) due to coil failure
	ED) drift of unknown source
	F) Artifacts detected with independent
	components analysis
	G) Hyperintensity of single slices
	H) Vertical strikes in the sagittal
	plane of the standard
	deviation map
	I) Data formatting issues

The order of the criteria is arbitrary.

#### 2.3.2. Image processing

Image processing was carried out according to our protocol (Esteban et al., 2020). First, we ran MRIQC with a Docker container of its latest version 22.0.1 (Listing 1 shows an example script). This version performs head motion estimation with AFNI (version 22.0.17; Cox, 1996), followed by brain extraction with SynthStrip (Hoopes et al., 2022) and several image registration tasks with ANTs (version 2.3.3.dev168-g29bdf; Avants et al., 2008). Since data were already BIDS compliant, no formatting or adaptation actions were required before running MRIQC. MRIQC generated one visual report per T1w image and BOLD scan, which author CP evaluated as part of the QC protocol described below. The panels presented in the visual report are specific to the modality, meaning that different visualizations are presented for an anatomical scan compared to a functional scan. Once all the visual reports had been evaluated as indicated below (Assessment of the unprocessed data), we executed fMRIPrep only on those subjects for which the T1w and at least one BOLD scan had passed the initial QC checkpoint. As for MRIQC, fMRIPrep could be directly run on the BIDS inputs using the corresponding Docker container at version 22.0.0 (see Supplementary material, section 4). As a result, fMRIPrep yielded preprocessed data and one individual QA/QC report per subject. Based on these individual reports, we established our second QC checkpoint, which was executed by author CP. The scripts we ran to execute MRIQC on the task fMRI data and fMRIPrep on the preprocessed data can be found in the Supplementary material, section 3.

**Listing 1:**
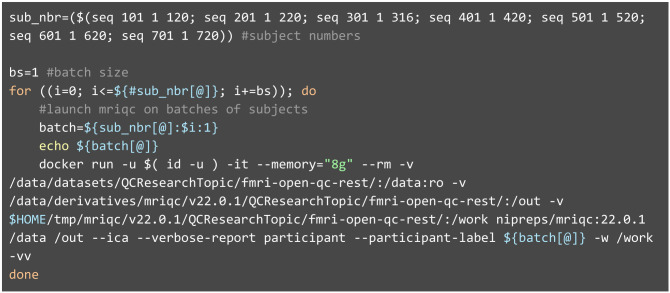
Execution of MRIQC with a Docker container. MRIQC follows the standards laid out by BIDS-Apps (Gorgolewski et al., 2017). As such, the command line using containers is composed of a preamble configuring Docker, the name of the specific Docker image (nipreps/mriqc:22.0.1), and finally, MRIQC's arguments. Because SynthStrip is a deep-learning-based approach, the brain masking step requires at least 8GB of memory (specified by the—memory flag).

#### 2.3.3. Assessment of the unprocessed data

Visualization of reports was performed on a 27” monitor. The reports corresponding to each BOLD scan were assessed first, following the reports' ordering of visualizations. Once the full report had been visualized, CP would return to specific sections of the report when a second assessment was necessary. Finally, author CP reported her QC assessment on a spreadsheet table (included in the Supplementary material), indicating which criteria led to exclusion. The exclusion criteria are described in detail in Section 2.4. A similar protocol was then applied for screening all reports corresponding to T1w images.

#### 2.3.4. Assessment of the minimally preprocessed data

Visualization of reports was performed on a 27” monitor. The reports corresponding to subjects that passed the previous checkpoint were screened one by one by CP. Author CP manually noted down the corresponding assessments on a spreadsheet table (included in the Supplementary material).

### 2.4. Assessment of quality aspects and exclusion criteria

Our exclusion criteria are all based on the visual inspection of the individual MRIQC and fMRIPrep reports, so they are all qualitative. Exclusion criteria are defined in reference to specific artifacts and qualitative aspects of BOLD and T1w images. Furthermore, we did not differentiate criteria for task and resting-state scans because our defined scope was not specific enough (e.g., lacking in objectives to determine whether some regions are of particular interest), except for the hyperintensity of single slices criterion. Each criterion is labeled for further reference in the document, the rater's notes, and the SOPs documents. Table 1 exhaustively lists the exclusion criteria based on the MRIQC visual report of BOLD data, Table 2 lists the criteria used to flag T1w data based on the MRIQC visual report, and Table 3 lists the exclusion criteria based on fMRIPrep visual reports. These tables are also cross-referenced with each criterion's label.

**Table 2:** T1w flagging criteria based on the MRIQC visual report.

QC of unprocessed T1w data based on the MRIQC visual report	J) Artifactual structures in the background
	K) Susceptibility distortion artifacts KA) Signal drop-out KB) Brain distortions
	L) Aliasing ghost
	M) Wrap-around that overlaps with the brain
	N)Data formatting issues
	O) Motion-related and Gibbs ringing
	P) Extreme intensity non-uniformity
	Q) Eye spillover

The order of the criteria is arbitrary.

**Table 3:** Resting-state and task exclusion criteria based on the fMRIPrep visual report.

QC of preprocessed data based on fMRIPrep visual report	R) Failure in normalization to MNI space
	S) Inaccurate brain mask
	T) Residual susceptibility distortion
	U) Error in brain tissue segmentation of T1w images
	V) Surface reconstruction problem
	W) Co-registration problem
	X) Regions identified for the extraction of nuisance regressors potentially cover neural signal sources

The order of the criteria is arbitrary.

#### 2.4.1. Exclusion criteria for unprocessed BOLD data assessed with MRIQC visual reports

##### 2.4.1.1. Artifactual structures in the background (Criterion A)

Because no BOLD signal originates from the air surrounding the head, the background should not contain visible structures. However, signals sourcing from the object of interest can spill into the background through several imaging processes, e.g., aliasing ghosts, spillover originating from moving and blinking eyes, or bulkhead motion. Structures in the background are most clearly noticeable in MRIQC's “background noise panel” view, but they are frequently detectable in the standard deviation map view. Structure in the background is not a problem in itself as it is situated outside of the brain; the issue is that the latter artifact is likely overflowing on the brain, thus compromising brain signal. The aliasing ghost is a particular case of spurious structures in the background, discussed in further detail in criterion C below. We classified under exclusion criteria A all other structures that did not correspond to an aliasing artifact. Supplementary Figure 1 shows several illustrative examples.

##### 2.4.1.2. Susceptibility distortion artifacts (B)

Susceptibility distortions are caused by B_0_ field non-uniformity (Hutton et al., 2002). Indeed, inserting an object in the scanner bore perturbs the nominal B_0_ field, which should be constant all across the FoV. Specifically, tissue boundaries produce steps of deviation from the nominal B_0_ field, which are larger where the air is close to tissues. Because of these deviations, the signal is recorded at locations slightly displaced from the sampling grid along the phase encoding axis leading to susceptibility distortions (Esteban et al., 2021). Susceptibility distortions manifest in two different ways on the BOLD average panel of the MRIQC visual report (Supplementary Figure 2): as signal drop-out, that is, a region where the signal vanishes (criterion BA), or as brain distortions (criterion BB). Signal drop-outs often appear close to brain-air interfaces, as explained below; these include ventromedial prefrontal cortex, the anterior part of the prefrontal cortex, and the region next to the ear cavities. Susceptibility distortion artifacts can be corrected by the susceptibility distortion correction implemented in fMRIPrep, provided that a field map associated with the BOLD image has been acquired and is correctly referenced in the dataset. This means that the presence of susceptibility distortions does not necessarily constitute an exclusion criterion. However, given the application scope of this paper, since no field maps were shared with the dataset and because we did not identify regions of little interest where these artifacts may be less detrimental, any signal drop-out observed resulted in the exclusion of the scan. In practice, legacy datasets without field maps can still be usable if researchers take adequate mitigation approaches (which also require rigorous QA/QC).

##### 2.4.1.3. Aliasing ghosts (C)

A ghost is a type of structured noise that appears as shifted and faintly repeated versions of the main object, usually in the phase encoding direction. They occur for several reasons, such as signal instability between pulse cycle repetitions or the particular strategy of echo-planar imaging to record the k-space during acquisition. Ghosts are often exacerbated by within-volume head motion. Sometimes they can be spotted in the BOLD average view of the MRIQC visual report, but they are more apparent in the background noise visualization. We excluded the scans for which ghosts were approximately the same intensity as the brain's interior in the background noise visualization. Supplementary Figure 3 compares an aliasing artifact that led to exclusion and one that did not.

##### 2.4.1.4. Wrap-around (D)

Wrap-around occurs whenever the object's dimensions exceed the defined field-of-view (FOV). It is visible as a piece of the head (most often the skull, in this dataset) being folded over on the opposite extreme of the image. We excluded subjects based on the observation of a wrap-around only if the folded region contained or overlapped the cortex. In the MRIQC visual report, the wrap-around can be spotted on the BOLD average, standard deviation map, and the background noise visualization. However, we found that the background noise visualization is the clearest to assess whether the folded region overlaps the brain (Supplementary Figure 4). Note that increasing the screen's brightness helps when looking for both aliasing ghosts and wrap-around overlapping the brain, as low brightness makes the artifacts harder to see.

##### 2.4.1.5. Assessment of time series with the carpet plot (E)

The carpet plot is a tool to visualize changes in voxel intensity throughout an fMRI scan. It works by plotting voxel time series in close spatial proximity so that the eye notes temporal coincidence (Power, 2017). Both MRIQC and fMRIPrep generate carpet plots segmented in relevant regions. One particular innovation of these carpet plots is that they contain a “crown” area corresponding to voxels located on a closed band around the brain's outer edge. As those voxels are outside the brain, we do not expect any signal there, meaning that if some signal is observed, we can interpret it as artifactual. Therefore, a strongly structured crown region in the carpet plot is a sign that artifacts are compromising the fMRI scan (Provins et al., 2022a). For example, motion peaks are generally paired with prolonged dark deflections derived from spin-history effects (criterion EA). Periodic modulations on the carpet plot indicate regular, slow motion, e.g., caused by respiration, which may also compromise the signal of interest (criterion EB). Furthermore, coil failures may be identifiable as a sudden change in overall signal intensity on the carpet plot and generally sustained through the end of the scan (criterion EC). In addition, sorting the rows (i.e., the time series) of each segment of the carpet plot such that voxels with similar BOLD dynamics appear close to one another reveals non-global structure in the signal, which is obscured when voxels are ordered randomly (Aquino et al., 2020). Thus, strongly polarized structures in the carpet plot suggest artifact influence (criterion ED). Supplementary Figure 5 illustrates the four types of carpet plot patterns. Finding temporal patterns similar in gray matter areas and simultaneously in regions of no interest (for instance, cerebrospinal fluid or the crown) indicates the presence of artifacts, typically derived from head motion. If the planned analysis specifies noise regression techniques based on information from these regions of no interest [which is standard and recommended (Ciric et al., 2017)], the risk of removing signals with neural origins is high, and affected scans should be excluded.

##### 2.4.1.6. Artifacts detected with independent components analysis (F)

MRIQC was run with the --ica argument, which generates an independent component decomposition using FSL MELODIC (version 5.0.11; Beckmann and Smith, 2004). Such techniques have been thoroughly described elsewhere (Griffanti et al., 2017). Components are easily screened with the specific visualization “ICA components” in the corresponding BOLD report, and each component is mapped on a glass brain with an indication of their frequency spectrum and their corresponding weight over time. One recurring artifactual family of components emerges when motion interacts with interleaved acquisition giving rise to the so-called spin-history effects. The spin-history effects appear as parallel stripes covering the whole brain in one direction (see Supplementary Figure 6). They are a consequence of the repetition time not being much larger than the T1 relaxation time in typical fMRI designs. This implies that the spins will not completely relax when the next acquisition starts.^1^ In addition, specific movements (e.g., rotation around one imaging axis, such as nodding) will exacerbate spin-history effects as slices will cut through the brain at different locations between consecutive BOLD time points. These two considerations combined mean that motion will produce spins with different excitation histories, and thus, the signal intensity will differ. Components showcasing parallel stripes concurring with slices in extreme poles of the brain or even across the whole brain are likely to capture these effects.

##### 2.4.1.7. Hyperintensity of single slices (G)

Above the carpet plot, MRIQC and fMRIPrep represent several time series to support the interpretation of the carpet. In particular, the slice-wise z-standardized signal average is useful for detecting sudden “spikes” in the average intensity of single slices of BOLD scans. When paired with the motion traces, it is possible to determine whether these spikes are caused by motion or by possible problems with the scanner (e.g., white-pixel noise). Spikes caused by motion typically affect several or all slices, while spikes caused by white-pixel noise affect only one slice and are generally more acute (see Supplementary Figure 7). White-pixel noise is generally caused by some small pieces of metal in the scan room or a loose screw on the scanner that accumulates energy and then discharges randomly. This creates broad-band RF noise at some point during the signal read-out, leading to one spot in the k-space with abnormally high intensity. In the image domain, it manifests as an abrupt signal intensity change in one slice at one time point. The problem is particularly acute for EPI scans because of all the gradient blipping during the read-out. For resting-state data, we discarded BOLD scans containing these spikes regardless of their physical origin (motion vs. white-pixel noise) because correlation analyses are likely biased by such peaks. Conversely, task data analyses are typically more robust to this particular artifact. Therefore the presence of only one or more relatively small spikes led to the scan being flagged for careful inspection after the preprocessing.

##### 2.4.1.8. Vertical strikes in the sagittal plane of the standard deviation map (H)

The sagittal view of the standard deviation map might show vertical strike patterns that extend hyperintensities through the whole sagittal plane (see Supplementary Figure 8). We excluded all images showcasing these patterns. Although we did not find an explanation of the mechanism behind this artifact, email conversations dating from 2017 seemed to point at an interaction between physiology and environmental issues in the scanning room that may affect the receiver coils.

##### 2.4.1.9. Data formatting issues (G)

As part of the NIfTI format (Cox et al., 2004), the file header contains metadata storing several relevant parameters, of which the orientation information is critical for the interpretability of the data. The orientation parameters indicate how the data matrix is stored on disk and enable visualizing rows and slices at the correct locations (Glen et al., 2020). However, mistakes may occur while recording information at the scanner, converting DICOM to NIFTI format, or during a subsequent processing step. Such mistakes result in the brain image not being correctly visualized and preprocessed, with axes either being flipped (e.g., the anterior part of the brain is labeled as posterior) or switched (e.g., axial slices are interpreted as coronal ones). These issues may render the dataset unusable, e.g., if the orientation information describing whether the data array has been recorded from left to right or right to left is lost. Examples are shown in Supplementary Figure 9.

#### 2.4.2. Criteria for flagging unprocessed T1w data based on the MRIQC visual report

Given our planned analysis, the T1w image will be used solely to guide the spatial alignment to the standard MNI152NLin2009cAsym template. In addition, surface reconstructions from the T1w image will guide the co-registration of structural and functional (BOLD) images in fMRIPrep. Since the latter preprocessing steps are relatively robust to structural images with mild artifacts, we did not impose exclusion criteria on the unprocessed T1w images. However, we annotated subjects with visible artifacts in the T1w images to ensure rigorous scrutinizing of spatial normalization and surface reconstruction outputs from fMRIPrep (if both modalities passed the first QC checkpoint with MRIQC). The explanation and the description of the criteria J to N are the same as their counterpart in Section 2.4.1 and are illustrated in Supplementary Figure 10.

##### 2.4.2.1. Motion-related and Gibbs ringing (O)

Large head motion during the acquisition of T1w images often expresses itself with the appearance of concentric ripples throughout the scan (see Supplementary Figure 10E). In the most subtle cases, motion-related ripples look similar to the fine lines generated by Gibbs ringing. The latter emerges as a consequence of the truncation of the Fourier series approximation and appears as multiple fine lines immediately adjacent and parallel to high-contrast interfaces. While Gibbs ringing is limited to the adjacency of sharp steps in intensity at tissue interfaces, the ripples caused by motion generally span the whole brain and are primarily visible in the sagittal view of MRIQC's mosaic views.

##### 2.4.2.2. Intensity non-uniformity (P)

Intensity non-uniformity is characterized by a smooth variation (low spatial frequency) of intensity throughout the brain caused by the stronger signal sensed in the proximity of coils. It is noticeable on the zoomed-in view on the T1w image (see Supplementary Figure 10F). Furthermore, intensity non-uniformity can be a problem for automated processing methods that assume a type of tissue [e.g., white matter (WM)] is represented by voxels of similar intensities across the whole brain. An extreme intensity non-uniformity can also be a sign of coil failure.

##### 2.4.2.3. Eye spillover (Q)

Eye movements may trigger the signal leakage from the eyes through the imaging axis with the lowest bandwidth (i.e., acquired faster), potentially overlapping signal from brain tissue. On data preserving facial features, the streak of noise is visible in the background at the levels of the eyes. However, because all the data in this study are openly shared after defacing (for privacy protection reasons), the signal around the face has been zeroed, leading to this leakage not being visible (Provins et al., 2022b). A strong signal leakage can, however, be noticeable on the zoomed-in view of the T1w image (see Supplementary Figure 10G for an example of the latter case).

#### 2.4.3. Exclusion criteria of pre-processed data based on fMRIPrep visual report

##### 2.4.3.1. Failure in normalization to MNI space (R)

Because the conclusions of the hypothetical analysis are based on data normalized to a standard template, the normalization must be successful. The fMRIPrep report contains a widget to assess the quality of the normalization to MNI space. The widget flickers between the MNI template and the individual's T1w image normalized to that template. To verify successful normalization, we assessed the correct alignment of the following structures (in order of importance): (1) ventricles, (2) subcortical regions, (3) corpus callosum, (4) cerebellum, and (5) cortical gray matter (GM). A misalignment of the ventricles, the subcortical regions, or the corpus callosum led to immediate exclusion. However, we were more lenient with the misalignment of cortical GM because volumetric (image) registration may not resolve substantial inter-individual differences (e.g., a sulcus missing in an individual's brain but typically present in the population of the template). Any extreme stretching or distortion of the T1w image also indicates a failed normalization.

##### 2.4.3.2. Inaccurate brain mask (S)

The brain mask computed from the T1w image is shown in the “brain mask and brain tissue segmentation of the T1w” panel under the anatomical section of the fMRIPrep visual report. The latter should closely follow the contour of the brain. An inaccurate brain mask presents “bumps” surrounding high-intensity areas of signal outside of the cortex (e.g., a mask including patches of the skull) and/or holes surrounding signal drop-out regions. Having an accurate brain mask makes the downstream preprocessing of an fMRI scan faster (excluding voxels of non-interest) and more accurate (less bias from voxels of non-interest). Consequently, it is important to discard subjects for which the brain mask is not well defined. Note that the brain mask plotted in the “brain mask and (anatomical/temporal) CompCor ROIs” panel under the functional section is not identical to the brain mask mentioned in this paragraph, as it is computed from the BOLD image. This mask must not leave out any brain area. Therefore, an exclusion criterion can be established when the mask intersects brain-originating signals.

##### 2.4.3.3. Residual susceptibility distortion (T)

For cases that were not excluded following criterion B, susceptibility distortions were evaluated with the fMRIPrep report after preprocessing. Any observation of susceptibility distortion artifacts led to the exclusion of the scan (see Supplementary Figure 11).

##### 2.4.3.4. Error in brain tissue segmentation of T1w images (U)

The panel “brain mask and brain tissue segmentation of the T1w” under the anatomical section of the fMRIPrep report shows contours delineating brain tissue segmentations overlaid on the T1w image. To confirm the good quality of the segmentation, we first verified that the pink contour accurately outlined the ventricles, whereas the blue contour followed the boundary between GM and WM. The first exclusion criterion was thus the inclusion of tissues other than the tissue of interest in the contour delineations. T1w scans showcasing a low signal-to-noise ratio because of thermal noise will present scattered misclassified voxels within piecewise-smooth regions (generally more identifiable in the WM and inside the ventricles). These scans were excluded except for images where these voxels are only present at subcortical structures (e.g., thalamus) or nearby tissue boundaries. In the latter case, the misclassification results from partial volume effects (i.e., indeed, such voxels contain varying fractions of two or more tissues). Supplementary Figure 12 illustrates the difference between individual dots caused by noise vs. partial volume effects.

##### 2.4.3.5. Surface reconstruction problem (V)

The WM surface (blue outline) and the pial surface (red outline) reconstructed with FreeSurfer [version 7.0.1, Fischl, 2012] are overlaid on the participant's T1w image, in the panel dedicated to surface reconstruction visualization under the anatomical section of the fMRIPrep report. Since the cerebellum and the brainstem are excluded from the surface reconstruction, the outlines will not include these areas. QC assessment of FreeSurfer outcomes is comprehensively covered elsewhere (e.g., White et al., 2018; Klapwijk et al., 2019), and fMRI studies using vertex-wise (surface) analyses should rigorously assess these surfaces. In this protocol, we only excluded data when the reconstructed surfaces were extremely inaccurate, which typically only happens in the presence of artifacts easily captured previously by MRIQC (Section 2.4.2).

##### 2.4.3.6. Co-registration problem (W)

The fMRIPrep report contains a widget to assess the accuracy of the alignment of BOLD runs into the individual's anatomical reference (co-registration). The widget flickers between the reference T1w image and the BOLD average co-registered onto it. Extracted brain surfaces' contours are represented as further anatomical cues. Here, we checked the alignment of image intensity edges and the anatomical landmarks (e.g., the ventricles and the corpus callosum) between the BOLD and the T1w images.

##### 2.4.3.7. Regions identified for the extraction of nuisance regressors potentially cover neural signal sources (X)

fMRIPrep calculates CompCor (Behzadi et al., 2007) nuisance regression time series to remove physiological and head motion artifacts from BOLD scans. Two families of CompCor methodologies are provided within the outputs: temporal CompCor (tCompCor) uses voxels presenting the highest temporal variability, and anatomical CompCor (aCompCor) extracts signal from regions of no interest (e.g., a conservative mask including core areas of the ventricles and the WM). fMRIPrep provides a panel to assess the adequacy of these regions from which CompCor will extract regressors (“brain mask and anatomical/temporal CompCor ROIs”). In addition to the masks corresponding to CompCor, the “crown” mask can also be assessed in this visualization. If the study plan prescribes using CompCor or brain-edge regressors, it is critical to exclude BOLD runs where any of these masks substantially overlap regions of interest.

## 3. Results

Following our predefined exclusion criteria, we excluded all the BOLD scans at the first QC checkpoint, except 4/151 for the resting-state subset and 1/30 for the task subset (97% of the subjects were excluded). The high exclusion rate was expected as this dataset had been preselected to contain data expressing a wide range of artifacts. In a standard dataset, the exclusion rate usually lays between 10 and 25% (Esteban et al., 2017). By far, the most common reason for exclusion was the presence of susceptibility distortion (exclusion criterion B). Other commonly found artifacts that met the exclusion criteria included aliasing ghost (C), problematic wrap-around (D), and structured carpet plots (E). The number of times each criterion has been cited as a reason for exclusion is reported in Supplementary Table 1. Moreover, 58/181 T1w images were flagged for thorough scrutinization of the normalization and the surface reconstruction outputs of fMRIPrep. One T1w image was exceptionally excluded based on the MRIQC visual report because of extreme motion-related ringing. An overview of how often a scan was flagged based on which criterion can be found in Supplementary Table 2. Out of the five subjects that passed the first QC checkpoint, two were excluded based on the inspection of the fMRIPrep visual reports for the presence of previously undetected signal drop-out. Some of our criteria did not result in the exclusion of data in this dataset: spin-history effects, failed normalization, problematic brain masks of either T1w or BOLD images, surface reconstruction problem, and failed co-registration.

### 3.1. QC of MRI data substantially relies on the background

**Figure 1:**
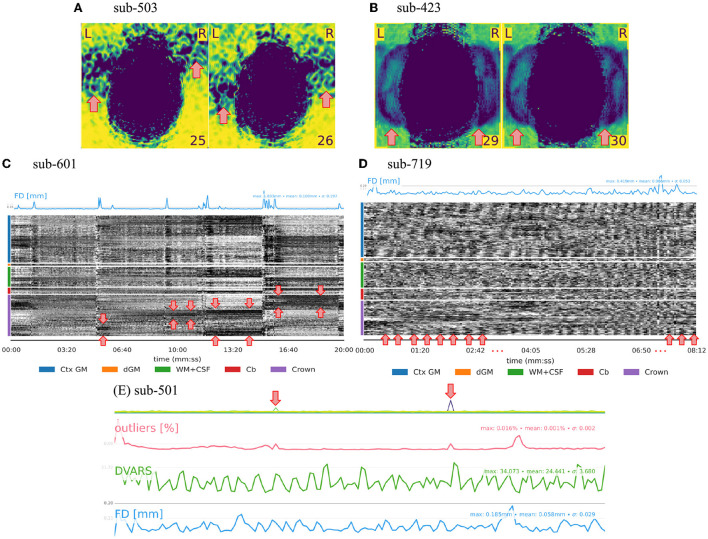
QA/QC of MRI data relies substantially on the background. Several exclusion criteria listed in Tables 1, 2 are based on the background. **(A)** Heavy structure in the background constitutes an exclusion criterion as the artifact likely extends inside the brain thus compromising signals of interest. **(B)** Aliasing ghosts appear as a faint and shifted copy of the brain in the background. **(C, D)** Since the crown comprises voxels outside the brain, the structure in the crown region of the carpet plot springs from artifacts. For example, two types of motion-related patterns can be distinguished. **(C)** Prolonged dark deflections are often caused by motion outbursts, visible as peaks in the framewise displacement (FD) trace. **(D)** Periodic fluctuations of intensity throughout the carpet plot can be attributed to periodic motion due to respiration. **(E)** The presence of sudden intensity change in a single slice can be attributed to white-pixel noise and constitutes an exclusion criterion.

The visual assessment of the “background noise” section of MRIQC reports helps unveil several artifactual structures suggesting further issues within the regions of interest (see Figure 1A). Aliasing ghosts that manifest as faint and shifted copies of the brain visible in the background are a particular type of structure in the background (see Figure 1B). Secondly, the background enclosed by the crown region plays an important role in detecting structure in the carpet plot. The influence of motion outbursts can be seen as prolonged dark deflections (see Figure 1C). Conversely, the presence of periodic modulation of the intensity can be attributed to periodic motion related to respiration (see Figure 1D). Thirdly, following the assumption that the slice-wise noise average on the background should be smooth, peaks in the single slices indicate some issue at the acquisition (i.e., white-pixel noise illustrated in Figure 1E). Overall, in adult MRI, no BOLD signal originates from the background, meaning that structures visible in the background come from artifacts. This consideration renders the background a convenient resource to assess MRI scans.

### 3.2. Setting QC checkpoints at several steps of the preprocessing is important

**Figure 2:**
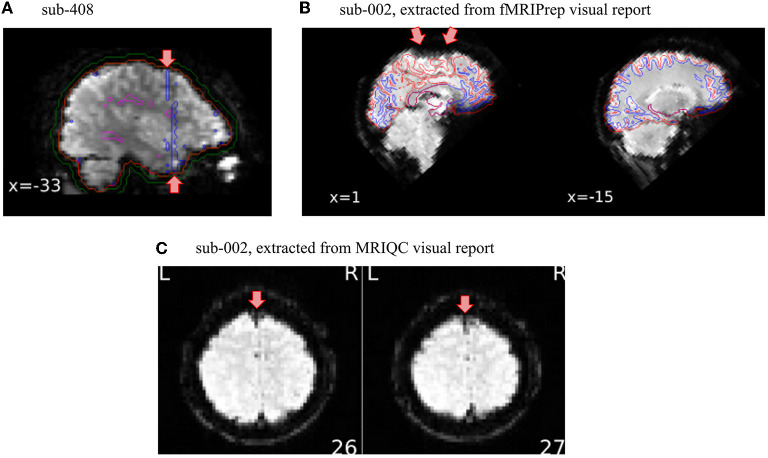
Setting QA/QC checkpoints at several steps of the preprocessing is important. Overlooking exclusion criteria while inspecting the visual reports can happen. Thus, having several QA/QC checkpoints set up along the preprocessing pipeline is valuable to catch those missed substandard scans. **(A)** In this particular case, the shape of the tCompCor mask looks suspiciously induced by an artifact, which led us to exclude this subject from further analysis. **(B)** This sagittal slice of the BOLD average presented in the fMRIPrep visual report clearly shows susceptibility distortion on the superior frontal cortex. This specific slice however did not appear in the MRIQC visual report. **(C)** The signal drop-out was furthermore more subtle on the axial slices, leading to an overlook of this artifact on the QA/QC checkpoint of unprocessed data.

In this protocol, we illustrate how we set up two QC checkpoints: one for unprocessed data using MRIQC visual report and one for minimally preprocessed data using fMRIPrep visual report. Only the data that survived the first QC checkpoint with MRIQC were run through fMRIPrep, illustrating how QC must drop data that meet exclusion criteria. The checkpoint leveraging fMRIPrep's visual report is important not only to capture problems in the processing of the data (e.g., failure in co-registration) but it also offers another opportunity to look at the data from different perspectives. To illustrate this point, we simulated a scenario where exclusion criteria were intendedly misapplied in the QC checkpoint based on MRIQC for one subject (sub-408), and as a result the dataset was inappropriately run through fMRIPrep. Figure 2A presents the tCompCor mask obtained for this subject, which suggests the presence of an artifact by its shape and its large overlap with the region of interest. These considerations justified the exclusion of the subject. Note furthermore that we did not detect that specific artifact in the MRIQC visual report (even after specifically looking out for it), illustrating the value of looking at the data using many different visualizations. Besides, viewing many slices cutting in several planes helps to not overlook exclusion criteria as illustrated in Figures 2B, C. Indeed, a signal drop-out that appeared very clearly on a specific sagittal slice (see Figure 2B) was more subtle to detect on axial slices (see Figure 2C). This specific sagittal BOLD average slice was displayed in the panel “Alignment of functional and anatomical MRI data (surface driven)” of the functional part of the fMRIPrep report, a panel for which the original purpose is to assess the quality of co-registration and not to visualize BOLD average. This reinforces again the importance of viewing the data from different perspectives.

### 3.3. Exclusion criteria depend on the particularities of the project

**Figure 3:**
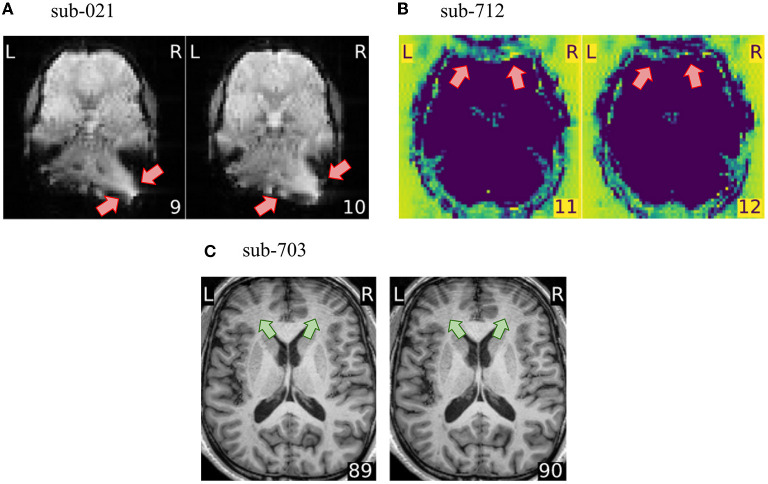
The exclusion criteria depend on the particularities of the project. **(A)** fMRIPrep can correct for susceptibility distortions when field maps are available. In this project, we however consider susceptibility distortion artifacts as exclusion criteria because no field maps were shared with the dataset. **(B)** A wrap-around overlapping the prefrontal cortex would not necessarily yield scan exclusion if the research question would focus on e.g., motor cortex. Our application scope has been defined as voxel-wise whole-brain fMRI analysis, thus this wrap-around is problematic. **(C)** Motion-related ringing on the T1w image does not constitute an exclusion criterion in our protocol, because the T1w is used solely for guiding the normalization and the co-registration. However, if the application scope would use surface-based analysis, this ringing would distort surface reconstruction.

How QA/QC is performed must be defined in close relation to the scope, goals, and approach of the project at hand. The first consideration is the types of data available. For example, the absence of field maps in the dataset led us to exclude a substantial portion of subjects that presented susceptibility distortion artifacts (see Figure 3A). Susceptibility distortion artifacts not only appeared as signal drop-outs or brain distortions, but they also interacted with head motion creating ripples that blurred the structure and destroyed contrast (see Supplementary Figure 2E). If field maps had been acquired and shared with the dataset, such artifacts could have been corrected by the susceptibility distortion correction run by fMRIPrep. This means that distortion present in the preprocessed data would grant exclusion at the corresponding checkpoint, but distortion present in the unprocessed data should not be considered an exclusion criterion. This example also highlights the importance of defining the exclusion criteria according to the placement of the QA/QC checkpoint within the research workflow. A further consideration is that the research question informs the regions where quality is most important. In the hypothetical scenario that a study investigates functional activity in the motor cortex, a wrap-around that affects the prefrontal cortex (see Figure 3B) would unlikely bias analyses limited to the region of interest. As such, it would not be considered an exclusion criterion in a study about the motor cortex. On the contrary, it would be very problematic for a study focusing on, e.g., decision-making. Finally, the planned analysis also determines the implementation of QA/QC protocols. In this paper, we did not exclude T1w images presenting motion-related ringing (see Figure 3C) because the application was scoped as a functional, voxel-wise analysis. If, instead, we would have set the application's scope as a vertex-wise (surface) analysis, then ringing on the T1w image would have granted exclusion, as the reconstructed brain surfaces from T1w images presenting the artifact would have been unreliable.

## 4. Discussion

We presented a QC protocol implemented on top of our previous fMRI analysis protocol (Esteban et al., 2020). We further restricted the scope of the planned analyses within standard whole-brain, voxel-wise models for both task and resting-state fMRI. Under such delineation of the application, we proposed two QC checkpoints: first, on the unprocessed data with MRIQC, and second, on the minimally preprocessed data with fMRIPrep. To fully reflect best practices, we only preprocessed the data corresponding to subjects for which the T1w image and at least one BOLD run had passed the first QC checkpoint. In this report, we also described the exclusion criteria that we believe would match the planned application and clearly remark that it is critical that researchers define these exclusion criteria in the most comprehensive way before the data are acquired (or accessed, in case of reusing existing data).

Here, we also restricted our protocol to describe QC decisions (i.e., excluding sub-standard data that risk biasing the final results). We did not describe relevant QA aspects and actions that can be triggered by QC outcomes because all data in the study were reused. Indeed, the outputs of QC should be leveraged to prevent quality issues from propagating through prospective acquisition. One example of how QA is limited in studies reusing data is the availability of field maps to correct susceptibility-derived distortions in BOLD images. Indeed, when field maps are available, fMRIPrep will run susceptibility distortion correction by default. However, no field maps were available in the dataset. Although we could have used fMRIPrep's “field map-less” approach to address susceptibility distortions, we decided such a decision would complicate the QC protocol description with an experimental, non-standard feature of fMRIPrep. A second QA aspect derived from the example dataset is the choice of the phase encoding direction. The phase encoding direction is generally the most limited in terms of bandwidth, and as a result, most artifacts propagate along that direction. For example, in the case of eye spillover, eye movements are likely to produce artifacts, thus selecting the phase encoding to occur along the anterior-to-posterior direction over left-to-right will produce a larger overlap of artifacts with the brain. In practice, if a particular task involves eye motion (e.g., blinking, saccade), the left–right direction could be the better choice if no other consideration conflicts regarding phase encoding.

One often overseen aspect of QA/QC protocols is establishing strategies to account for raters' reliability. Indeed, intra-rater variability and drifts are strongly driven by the protocol implementation settings (e.g., changing the size of the screen or other screen technical capabilities), training, and attrition. Raters' training is particularly relevant, and it originates systematic differences in how QA/QC criteria are applied over the time span of the project. Therefore, it is critical to use mitigation strategies like randomly selecting a few earlier reports for re-evaluation or annotating subjects one is uncertain about and returning to it later in the QC process. Similarly, the implementation of QA/QC protocols must also plan for multiple raters and anticipate a plan to counter inter-rater variabilities and drifts idiosyncratic to each of them (e.g., defining a training program with specific examples, inter-rater “calibration”, etc.). Learned insights can be transferred in several ways: 1. from other subjects that expressed the artifact more clearly, 2. from examining the report of another modality of the same subject, 3. from a more senior rater, or 4. from visual inspection of other tools' output. For example, if the brain is not perfectly aligned with the imaging axes, the space between the cerebellum and the temporal lobe at the basal part of the brain appears bigger on one side of the other on axial slices. Inexperienced raters may interpret that some artifact occurred, although, in fact, the image is just visualized with some obliquity with respect to the sagittal plane. This misinterpretation would be more likely for BOLD images, as this might look like a single-sided signal drop-out.

A fundamental aspect of a robust QC protocol we have showcased is its funneling design. At every QC checkpoint, we must pre-establish some exclusion criteria that will result in dropping sub-standard data. For datasets limited in sample size, excluding data may reduce the power of the study below the planned estimation. More generally, even when the analysis plan anticipated some data replacement measures for data dropped at the earlier QC checkpoints, excluding data increases the costs of the study (in terms of scan time, subject time, etc.). In this case, real-time QA/QC (that is, during the acquisition or immediately after) is a promising strategy to minimize data exclusion and replacement costs (Heunis et al., 2020). Therefore, establishing these criteria will present the researcher with the challenge of striking an appropriate balance between being excessively stringent (and therefore, discarding too many images) and too lenient to the point that results are not reliable. For this reason, it is important to establish QC criteria from the perspective of all the QC checkpoints in the pipeline and to ensure the best trade-off. When developing this manuscript, we understood that setting the scope to “whole-brain voxel-wise” analyses would allow more flexible QC criteria for the T1w images at the MRIQC step and only mark borderline images for a more rigorous screening after the second QC checkpoint. Conversely, we also discovered some artifacts in the fMRIPrep visual report that could have been spotted in the MRIQC visual report of the same participant. Going back to the MRIQC visual report, we could understand why this detail escaped us at the first iteration and learn from our mistake. Therefore, layering QC checkpoints is critical to ensure the robustness of the whole protocol.

### 4.1. Limitations and deviations from our standard QC protocols

Several limitations stem from the specifics of the dataset used in this study. First, we could not take advantage of the MRIQC group report, in which the IQMs extracted from all images in a dataset are presented in scatter plots, because this dataset was composed from multiple sources, which makes these reports hard to interpret without “harmonizing” the IQMs. On a single-site dataset, we would use the MRIQC group report to spot outliers in the IQMs distributions and double-check their corresponding visual reports for exclusion criteria. Second, we used the same exclusion criteria for the resting-state and task fMRI data, with the exception of criterion G (hyperintensity of single slices). In this particular case, we excluded resting-state runs showcasing G because this artifact will likely introduce correlations in the data that will potentially be interpreted as functional connections in such analyses. Conversely, models typically applied for analyzing task paradigms are generally more resilient to biases introduced by these hyperintensities. Third, the quality of the T1w images may have been overestimated because the data are defaced. As we explored in a recent pilot study (Provins et al., 2022b) defacing, though necessary to protect participants' privacy when sharing data publicly, likely biases manual QA/QC of anatomical images. One of our conclusions was that defaced images were perceived as having a better quality overall. Fourth, as a result of the QC data funnel mentioned above, the number of subjects for which we assessed the visual reports of fMRIPrep was severely limited to only the five out of 181 that passed the first QC checkpoint with MRIQC. The number of subjects successfully passing the first checkpoint would have been much higher if available field maps had been available within each subject's data, considering that criterion B (susceptibility distortions) was by large the top one criterion that granted exclusion of images. Lastly, the scope of the study was limited to describing the protocols and communicating our assessments. Although it would have been of interest to evaluate inter-rater and intra-rater variabilities, the settings were not adequate to address such questions. Indeed, with such a high (and expected) exclusion rate, in addition to the task of identifying as many subpar images as possible, both sources of variability in quality annotations will be certainly minimal. We explored such variabilities in Provins et al. (2022b) and we are currently extending the study with the pre-registration of a larger scale analysis (Provins et al., 2022c).

## 5. Conclusion

Establishing appropriate QC protocols adds to the list of practices conducive toward reliable neuroimaging workflows. Moreover, standardizing these protocols is critical to minimize intra-, and inter-rater, as well as intra- and inter-laboratories variabilities, thereby achieving consensus regarding QA/QC across researchers and opening ways to consistently train machine agents to automate the process. Therefore, the research topic in which this work is framed is a timely initiative pursuing such goals. We demonstrated the implementation of a QC protocol in a standard functional MRI analysis workflow at two checkpoints: (i) assessing the unprocessed data (with MRIQC) and (ii) assessing minimally preprocessed data (with fMRIPrep). We expect this thorough description of the QC protocol and associated data exclusion criteria built upon this research topic's initiative to promote best practices in QA/QC and help researchers implement their protocols for functional MRI more effectively.

## Data availability statement

The data used in this study are publicly available at open-source repositories under permissive licenses, and a single collection with all the original data is accessible at https://osf.io/qaesm/ under the CC-BY Attribution 4.0 International license. All MRIQC's visual reports referenced throughout the manuscript are openly available under the terms of the CC0 license at https://mriqc.s3.amazonaws.com/index.html#FrontiersQC/. Correspondingly, all fMRIPrep's visual reports are openly available under the CC0 license at https://fmriprep.s3.amazonaws.com/index.html#FrontiersQC/. The SOPs repository generated from the MRIQC-SOPs template can be found at https://github.com/frontiers-qc-sops and is distributed under a CC0 license. Correspondingly, a human-readable documentation website is generated with every update to the SOPs, accessible at http://www.axonlab.org/frontiers-qc-sops/. For archival purposes, the exact version that accompanies this manuscript is available with https://doi.org/10.5281/zenodo.7221547.

## Ethics statement

Ethical review and approval was not required for the study on human participants in accordance with the local legislation and institutional requirements. Written informed consent for participation was not required for this study in accordance with the national legislation and the institutional requirements.

## Author contributions

Conceptualization, funding acquisition, project administration, and supervision: OE. Data curation and visualization: CP. Methodology and writing: CP, OE, and SS. Resources: PH and OE. All authors reviewed and edited the manuscript. All authors contributed to the article and approved the submitted version.

## Footnotes

^1^ https://imaging.mrc-cbu.cam.ac.uk/imaging/CommonArtefacts

## References

[B1] Alexander-Bloch A. Liv Clasen M. S. Lisa Ronan F. L. Jay Giedd A. R. (2016). Subtle in-scanner motion biases automated measurement of brain anatomy from *in vivo* MRI. Hum. Brain Map. 37, 2385–2397. 10.1002/hbm.23180 27004471 PMC5110234

[B2] Alfaro-Almagro F. Mark Jenkinson N. K. B. Jesper L. R. Andersson L. G. Gwenaëlle Douaud S. N. S. (2018). Image processing and quality control for the first 10,000 brain imaging datasets from UK Biobank. Neuroimage 166, 400–424. 10.1016/j.neuroimage.2017.10.034 29079522 PMC5770339

[B3] Aquino K. M. Ben D. Fulcher L. P. Kristina Sabaroedin A. F. (2020). Identifying and removing widespread signal deflections from FMRI data: rethinking the global signal regression problem. NeuroImage 212, 116614. 10.1016/j.neuroimage.2020.116614 32084564

[B4] Avants B. B. Epstein C. L. Grossman M. Gee J. C. (2008). Symmetric diffeomorphic image registration with cross-correlation: evaluating automated labeling of elderly and neurodegenerative brain. Med. Image Anal. 12, 26–41. 10.1016/j.media.2007.06.004 17659998 PMC2276735

[B5] Beckmann C. F. Smith S. M. (2004). Probabilistic independent component analysis for functional magnetic resonance imaging. IEEE Trans. Med. Imag. 23, 137–152. 10.1109/TMI.2003.822821 14964560

[B6] Behzadi Y. Khaled Restom J. L. Liu T. T. (2007). A component based noise correction method (CompCor) for BOLD and perfusion based FMRI. NeuroImage 37, 90–101. 10.1016/j.neuroimage.2007.04.042 17560126 PMC2214855

[B7] Biswal B. B. Mennes M. Zuo X. N. Gohel S. Kelly C. Smith S. M. . (2010). Toward discovery science of human brain function. Proc. Natl. Acad. Sci 107, 4734–4739. 10.1073/pnas.0911855107 20176931 PMC2842060

[B8] Ciric R. Thompson W. H. Lorenz R. Goncalves M. MacNicol E. E. Markiewicz C. J. . (2022). TemplateFlow: FAIR-sharing of multi-scale, multi-species brain models. Nat. Meth. 19, 1568–1571. 10.1038/s41592-022-01681-2 36456786 PMC9718663

[B9] Ciric R. Wolf D. H. Power J. D. Roalf D. R. Baum G. L. Ruparel K. . (2017). Benchmarking of participant-level confound regression strategies for the control of motion artifact in studies of functional connectivity. NeuroImage 154, 174–187. 10.1016/j.neuroimage.2017.03.020 28302591 PMC5483393

[B10] Cox R. W. (1996). AFNI: software for analysis and visualization of functional magnetic resonance neuroimages. Comput. Biomed. Res. 29, 162–173. 10.1006/cbmr.1996.0014 8812068

[B11] Cox R. W. Ashbruner J. Breman H. Fissell K. Haselgrove C. Holmes C. J. (2004). “A (Sort of) new image data format standard: NIfTI-1,” in 10th Annual Meeting of the Organization for Human Brain Mapping (Budapest).

[B12] Di Martino A. Yan C. G. Li Q. Denio E. Castellanos F. X. Alaerts K. . (2014). The autism brain imaging data exchange: towards a large-scale evaluation of the intrinsic brain architecture in autism. Mol. Psychiatry 19, 659–667. 10.1038/mp.2013.78 23774715 PMC4162310

[B13] Ducharme S. Albaugh M. D. Nguyen T. V. Hudziak J. J. Mateos-Pérez J. M. Labbe A. . (2016). Trajectories of cortical thickness maturation in normal brain development—the importance of quality control procedures. NeuroImage 125, 267–279. 10.1016/j.neuroimage.2015.10.010 26463175 PMC4691414

[B14] Esteban O. Adebimpe A. Markiewicz C. J. Goncalves M. Blair R. W. Cieslak M. . (2021). “The bermuda triangle of D- and f-MRI sailors—software for susceptibility distortions,” in 27th Annual Meeting of the Organization for Human Brain Mapping (OHBM). p. 1653. 10.31219/osf.io/gy8nt

[B15] Esteban O. Ciric R. Finc K. Blair R. W. Markiewicz C. J. Moodie C. A. . (2020). Analysis of task-based functional MRI data preprocessed with fMRIPrep. Nat. Prot. 15, 2186–2202. 10.1038/s41596-020-0327-3 32514178 PMC7404612

[B16] Esteban O. Daniel Birman M. S. Oluwasanmi O. Koyejo R. A. P. Gorgolewski K. J. (2017). MRIQC: advancing the automatic prediction of image quality in MRI from unseen sites. PLoS ONE 12, e0184661–e0184661. 10.1371/journal.pone.0184661 28945803 PMC5612458

[B17] Esteban O. Markiewicz C. J. Blair R. W. Moodie C. A. Isik A. I. Erramuzpe A. . (2019). fMRIPrep: a robust preprocessing pipeline for functional MRI. Nat. Meth. 16, 111–116. 10.1038/s41592-018-0235-4 30532080 PMC6319393

[B18] Esteban O. Poldrack R. A. Gorgolewski K. J. (2018). “Improving out-of-sample prediction of quality of MRIQC,” in Intravascular Imaging and Computer Assisted Stenting and Large-Scale Annotation of Biomedical Data and Expert Label Synthesis. *Lect. Notes Comput. Sci*. p. 190–99. 10.1007/978-3-030-01364-6_21

[B19] Fischl B. (2012). FreeSurfer. NeuroImage 62, 774–781. 10.1016/j.neuroimage.2012.01.021 22248573 PMC3685476

[B20] Fonov V. S. Evans A. C. McKinstry R. C. Almli C. R. Collins D. L. (2009). Unbiased non-linear average age-appropriate brain templates from birth to adulthood. NeuroImage 47, S102. 10.1016/S1053-8119(09)70884-5

[B21] Garcia M. Dosenbach N. Kelly C (2022). BrainQCNet: a deep learning attention-based model for multi-scale detection of artifacts in brain structural MRI scans. bioRxiv. 10.1101/2022.03.11.483983 PMC12290559 40800355

[B22] Glen D. R. Taylor P. A. Buchsbaum B. R. Cox R. W. Reynolds R. C. (2020). Beware (Surprisingly Common) left–right flips in your MRI data: an efficient and robust method to check MRI dataset consistency using AFNI. Front. Neuroinform. 14, 18. 10.3389/fninf.2020.00018 32528270 PMC7263312

[B23] Gorgolewski K. J. Auer T. Calhoun V. D. Craddock R. C. Das S. Duff E. P. . (2016). The brain imaging data structure, a format for organizing and describing outputs of neuroimaging experiments. Sci. Data 3, 160044. 10.1038/sdata.2016.44 27326542 PMC4978148

[B24] Gorgolewski K. J. Fidel Alfaro-Almagro T. A. Pierre Bellec M. C. Mallar Chakravarty M. N. W. C. (2017). BIDS apps: improving ease of use, accessibility, and reproducibility of neuroimaging data analysis methods. PLOS Comp. Bio. 13, e1005209. 10.1371/journal.pcbi.1005209 28278228 PMC5363996

[B25] Griffanti L. Douaud G. Bijsterbosch J. Evangelisti S. Alfaro-Almagro F. Glasser M. F. . (2017). Hand classification of FMRI ICA noise components. NeuroImage 154, 188–205. 10.1016/j.neuroimage.2016.12.036 27989777 PMC5489418

[B26] Heunis S. Rolf Lamerichs S. Z. Cesar Caballero-Gaudes J. F. A. J. Bert Aldenkamp M. B. (2020). Quality and denoising in real-time functional magnetic resonance imaging neurofeedback: a methods review. Hum. Brain Map. 41, 3439–3467. 10.1002/hbm.25010 32333624 PMC7375116

[B27] Hoopes A. Mora J. S. Dalca A. V. Fischl B. Hoffman M. (2022). SynthStrip: skull-stripping for any brain image. NeuroImage 260, 119474. 10.1016/j.neuroimage.2022.119474 35842095 PMC9465771

[B28] Hutton C. Andreas Bork O. J. Ralf Deichmann J. A. Turner R. (2002). Image distortion correction in FMRI: a quantitative evaluation. NeuroImage 16, 217–240. 10.1006/nimg.2001.1054 11969330

[B29] Keshavan A. Esha Datta I. M. M. Christopher R. Madan K. J. Henry R. G. (2018). Mindcontrol: a web application for brain segmentation quality control. NeuroImage 170, 365–372. 10.1016/j.neuroimage.2017.03.055 28365419

[B30] Keshavan A. Jason D. Yeatman A. R. (2019). Combining citizen science and deep learning to amplify expertise in neuroimaging. Front. Neuroinform. 13, 29. 10.3389/fninf.2019.00029 31139070 PMC6517786

[B31] Klapwijk E. T. Van De Kamp F. Van Der Meulen M. Peters S. Wierenga L. M. (2019). Qoala-T: a supervised-learning tool for quality control of freesurfer segmented MRI data. NeuroImage 189, 116–129. 10.1016/j.neuroimage.2019.01.014 30633965

[B32] Marcus D. S. Harms M. P. Snyder A. Z. Jenkinson M. Wilson J. A. Glasser M. F. . (2013). Human connectome project informatics: quality control, database services, and data visualization. NeuroImage Map. Connectome 80, 202–219. 10.1016/j.neuroimage.2013.05.077 23707591 PMC3845379

[B33] Markiewicz C. J. Gorgolewski K. J. Feingold F. Blair R. Halchenko Y. O. Miller E. . (2021). The OpenNeuro Resource for Sharing of Neuroscience Data. eLife 10, e71774. 10.7554/eLife.71774.sa2 34658334 PMC8550750

[B34] Mortamet B. Bernstein M. A. Jack C. R. Jr. Gunter J. L. Ward C. Britson P. J. . (2009). Automatic quality assessment in structural brain magnetic resonance imaging. Magn. Reson. Med. 62, 365–372. 10.1002/mrm.21992 19526493 PMC2780021

[B35] Power J. D. (2017). A simple but useful way to assess FMRI scan qualities. NeuroImage 154, 150–158. 10.1016/j.neuroimage.2016.08.009 27510328 PMC5296400

[B36] Power J. D. Barnes K. A. Snyder A. Z. Schlaggar B. L. Petersen S. E. (2012). Spurious but systematic correlations in functional connectivity MRI networks arise from subject motion. NeuroImage 59, 2142–2154. 10.1016/j.neuroimage.2011.10.018 22019881 PMC3254728

[B37] Provins C. Alemán-Gómez Y. Cleusix M. Jenni R. Richiardi J. Hagmann P. . (2022b). Defacing biases manual and automated quality assessments of structural MRI with MRIQC,” in 28th Annual Meeting of the Organization for Human Brain Mapping (OHBM) (Glasgow). p. WTh566. 10.31219/osf.io/8mcyz

[B38] Provins C. Alemán-Gómez Y. Richiardi J. Poldrack R. A. Hagmann P. Esteban O. (2022c). “Defacing biases in manual and automatic quality assessments of structural MRI with MRIQC,” in Peer Community in Registered Reports (Registered Report Under Consideration Toward Stage 1).

[B39] Provins C. Markiewicz C. J. Ciric R. Goncalves M. Caballero-Gaudes C. Poldrack R. A. Hagmann P. Esteban O. (2022a). Quality Control and nuisance regression of fMRI, looking out where signal should not be found. Proc. Intl. Soc. Mag. Reson. Med. 31, (ISMRM), pp. 2683. 10.31219/osf.io/hz52v

[B40] Shehzad Z. Giavasis S. Li Q. Benhajali Y. Yan C. Yang Z. . (2015). The preprocessed connectomes project quality assessment protocol-A resource for measuring the quality of MRI data. Front. Neurosci. Conf. Neuroinformatics. 10.3389/conf.fnins.2015.91.00047

[B41] White T. Jansen P. R. Muetzel R. L. Sudre G. El Marroun H. Tiemeier H. . (2018). Automated quality assessment of structural magnetic resonance images in children: comparison with visual inspection and surface-based reconstruction. Hum. Brain Map. 39, 1218–1231. 10.1002/hbm.23911 29206318 PMC6866370

[B42] Zalesky A. Fornito A. Cocchi L. Gollo L. L. van den Heuvel M. P. Breakspear M. (2016). Connectome sensitivity or specificity: which is more important? NeuroImage 142, 407–420. 10.1016/j.neuroimage.2016.06.035 27364472

