# Supplementary material for: Quality control in functional MRI studies with MRIQC and fMRIPrep
Source: Front Neuroimaging. 2023 Jan 12;1:1073734. doi: 10.3389/fnimg.2022.1073734 (PMC10406249; doi:10.3389/fnimg.2022.1073734)
Supplement: Supplementary file 1 [file Data_Sheet_1.pdf]

Supplementary Material

1 Illustrating QC criteria

This section is dedicated to illustrate representative examples of each QC item in Table 1, Table 2 and Table 3. Furthermore, when applicable we contrasted the exclusion criteria with an instance where the same artifact did not lead to exclusion. The artifact yielding exclusion are pointed using red arrows, while the artifact not yielding exclusion are pointed using green arrows.

1.1 Exclusion criteria for unprocessed BOLD data assessed with MRIQC visual reports

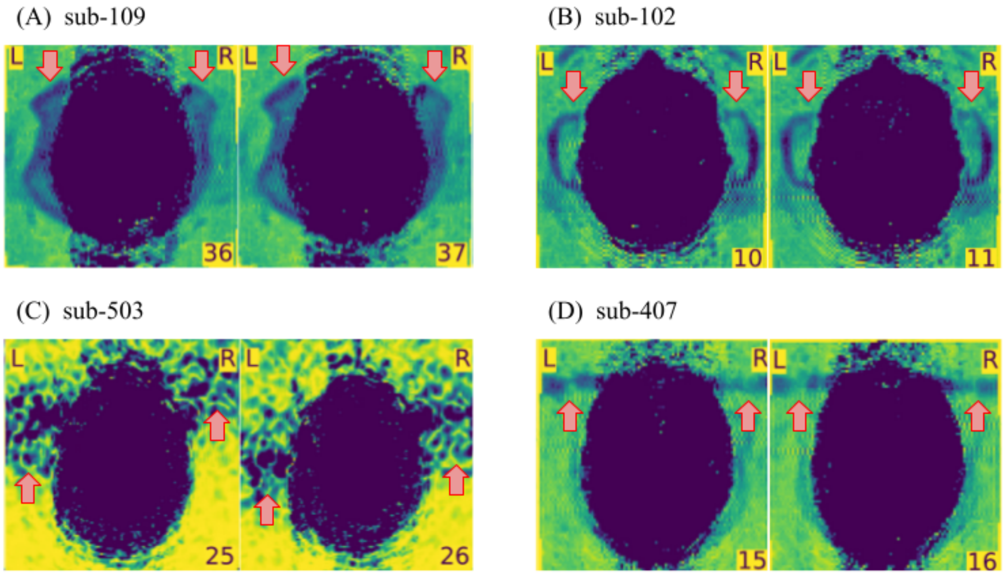

Figure S1. Artifactual structures in the background. Several types of structure were observed in the background: a cushion-like structure **(A)**, headphones **(B)**, heavy local perturbation **(C)**, strike **(D)**. All those patterns were visible in the standard map deviation as well.

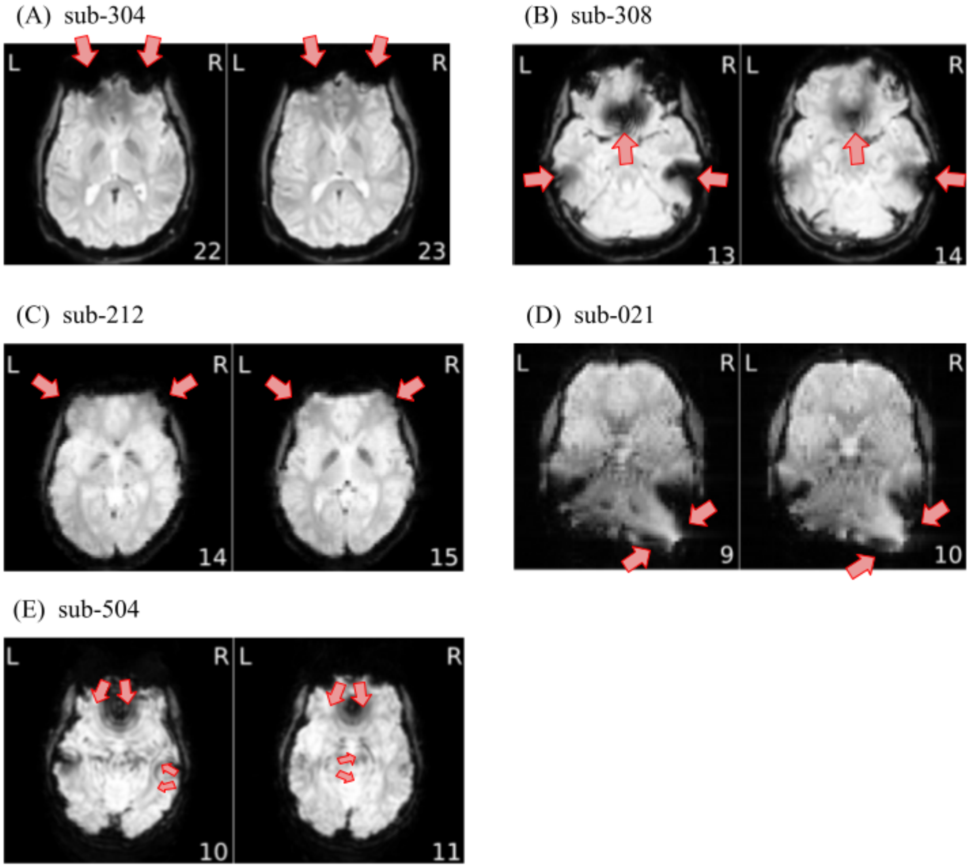

Figure S2. Susceptibility distortion artifacts. Susceptibility distortion artifacts manifest in two different ways: signal drop-outs (criterion BA) or brain distortions (criterion BB). Signal drop-outs were observed mostly at three different locations in this dataset : the anterior part of the prefrontal cortex **(A)**, the ventro-medial prefrontal cortex **(B)**, and next to the ear cavities **(B)**. Those regions are situated close to interfaces with air. Brain distortions typically appear as abnormal stretching of the prefrontal cortex **(C)**, or the occipital lobe **(D)**. Susceptibility distortion can also interact with head motion creating ripples that blurs the structure and destroys contrast **(E)**.

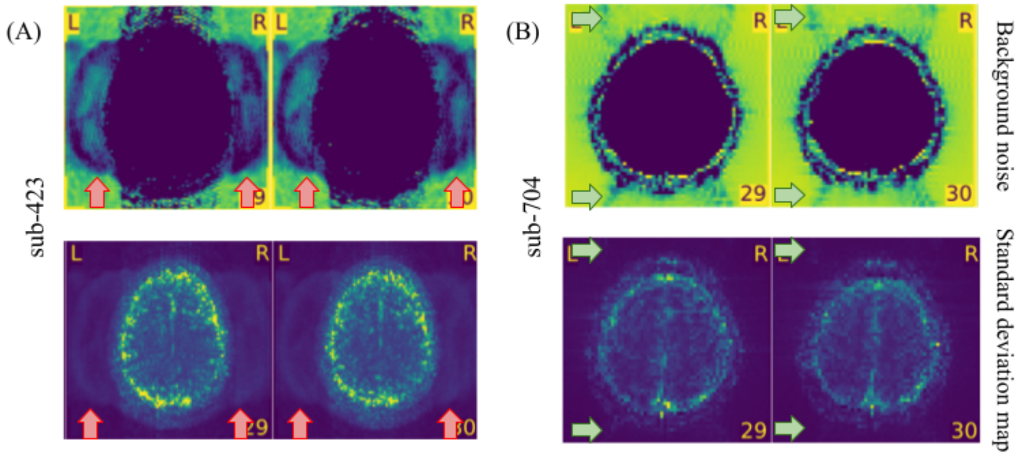

Figure S3. Aliasing ghosts. (A) Aliasing ghosts are visible as repetitions of the brain shape in the background noise and the standard deviation map. Because the intensity of the aliasing ghost is similar to the intensity of the brain on the background noise visualisation, this subject is thus excluded (criterion C). (**B)** The aliasing ghost is only faintly visible in the background noise, hence we would not exclude this subject for aliasing ghost.

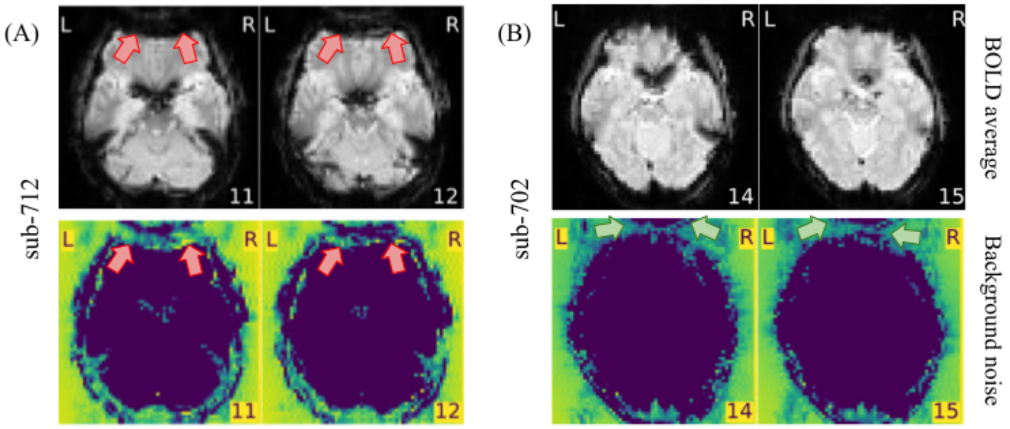

Figure S4. Problematic wrap-around. (A) The reflected skull overlaps with the prefrontal cortex; this subject thus abides by the exclusion criterion D. (**B)** The reflected skull does not overlap with the brain, hence exclusion criterion D does not apply here.

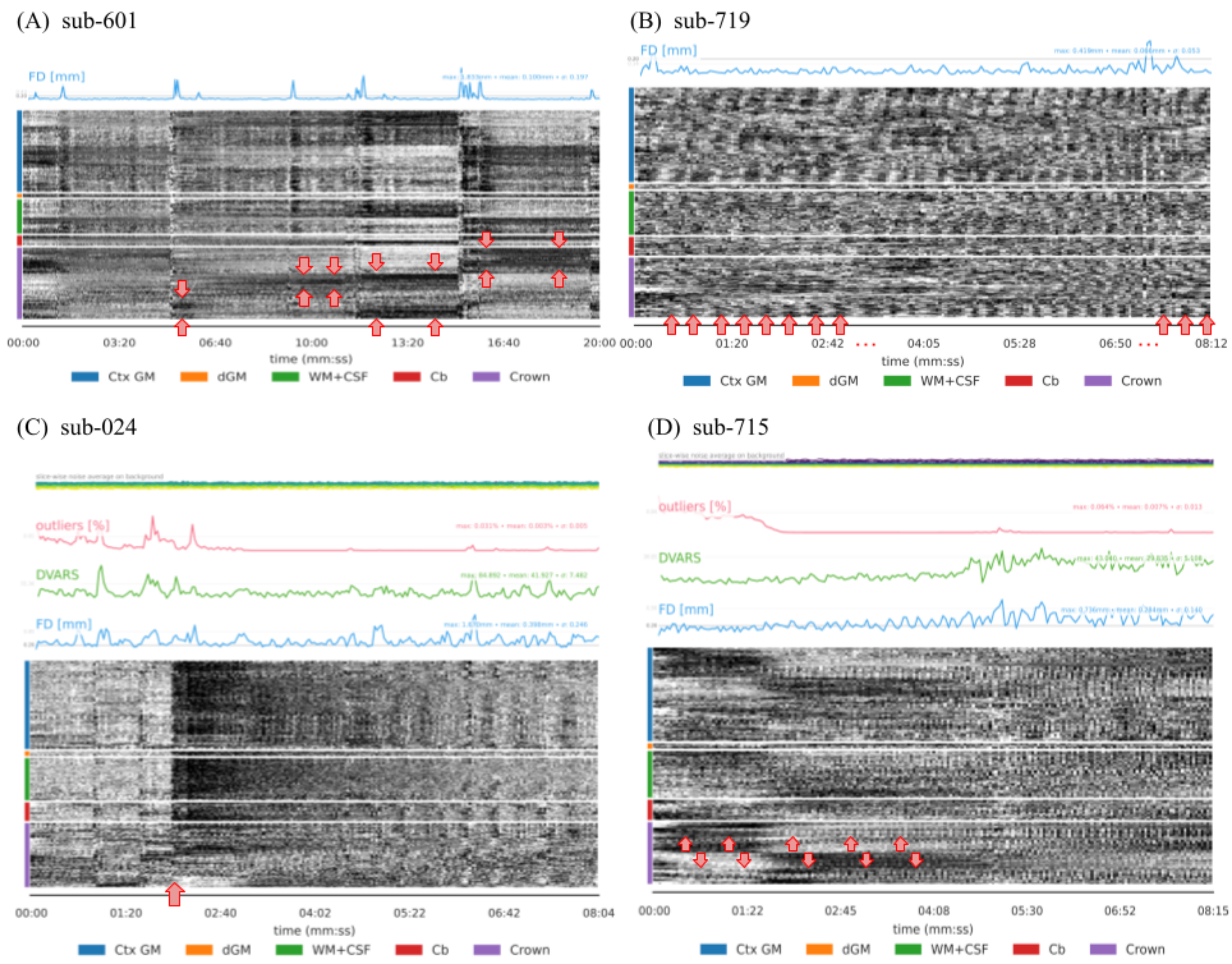

Figure S5. Assessment of time series with the carpet plot. The crown region of the carpet plot comprises voxels outside the brain. As such, structure in the crown can be interpreted as artifactual, and thus corresponds to an exclusion criteria. **(A)** Motion outbursts, visible as peaks in the framewise displacement (FD) trace, are often paired with prolonged dark deflections. **(B)** Periodic modulations are indicative of regular, slow motion, e.g., caused by respiration. **(C)** An abrupt change in overall signal intensity that is not paired with motion peaks can be attributed to coil failure. **(D)** A strong polarized structure revealed by the clustering of carpet plot rows also suggests that artifacts mitigate the signal of interest.

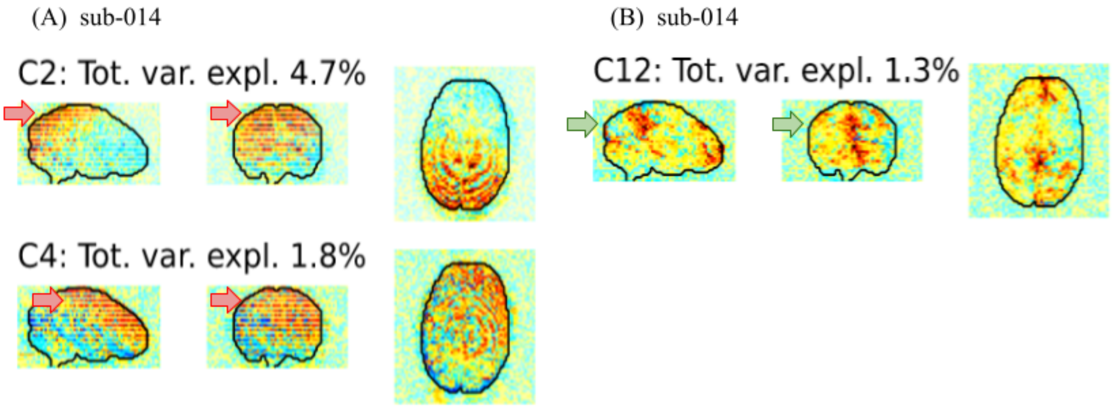

Figure S6. Artifacts detected with independent components analysis. (A) The presence of stripes on those components indicates spin-history effect. **(B)** As a comparison, this component, which is not corrupted by spin-history effects, does not express these stripes patterns.

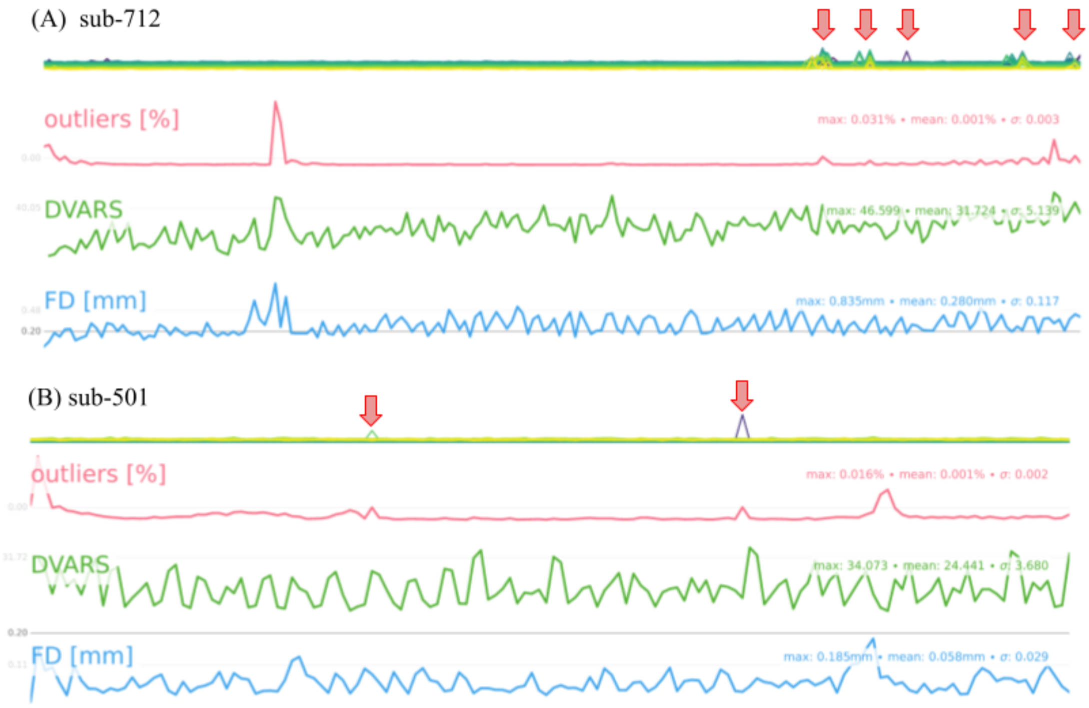

Figure S7. Hyperintensity of single slices. A) Motion-related peaks typically affect several or all slices. **B)** On the other hand, peaks in a single slice are associated with white-pixel noise. Because those two scans are resting-state data, hence are likely to be used for correlation analysis, the presence of peaks, regardless of their origin, yielded scans exclusion (criterion G).

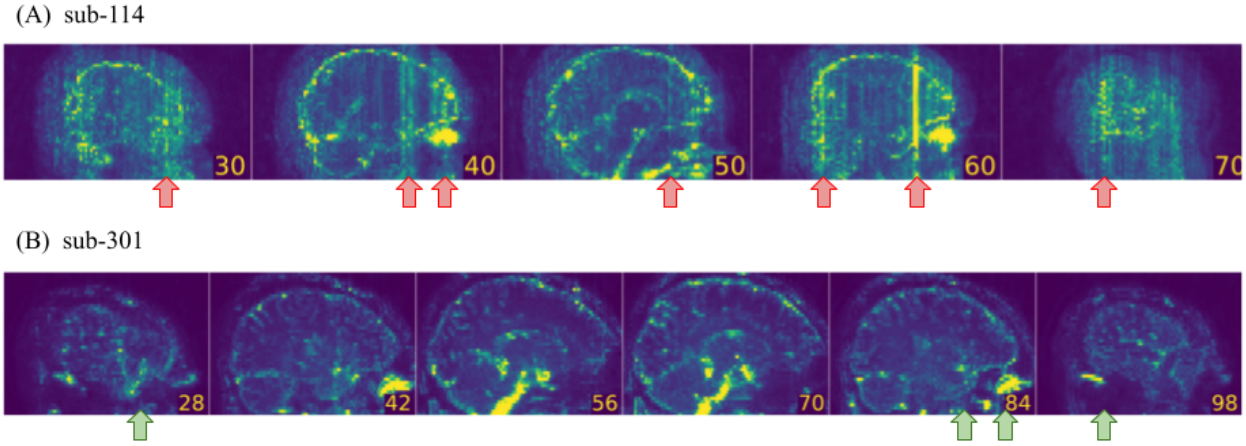

Figure S8. Vertical strikes in the sagittal plane of the standard deviation map. (A) High standard deviation strikes are extending hyperintensities along the whole sagittal plane (criterion H). (**B)** For reference, a subject for which the sagittal plane of the stp map is of good quality is illustrated. No vertical strikes are visible; the hyperintensities remain localized.

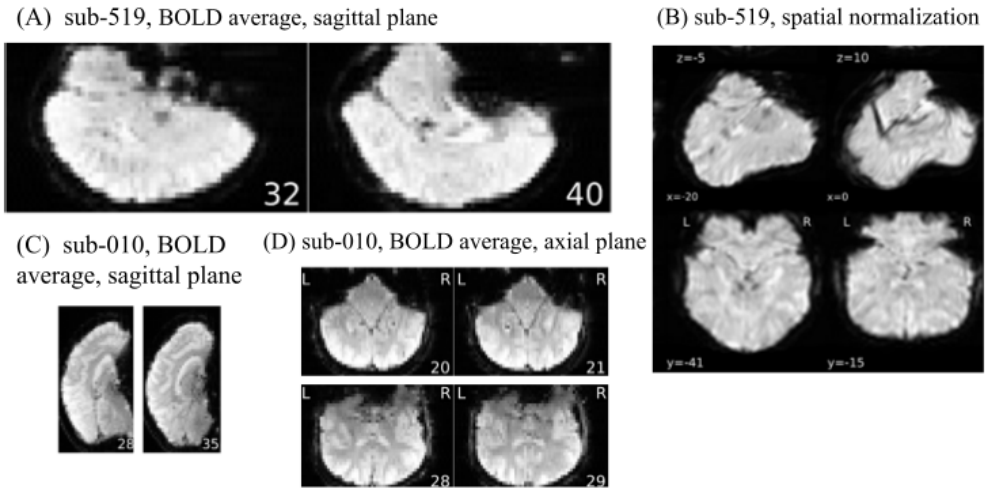

Figure S9. Data formatting issues. Header issues result in the BOLD average being wrongly visualized with axes being either flipped, e.g. the anterior part of the brain is labeled as posterior **(A)**, or switched, e.g. axial slices are interpreted as sagittal ones **(C, D)**. Header issues can additionally lead to complete failure in preprocessing, e.g completely distorted normalized map **(B)**.

1.2 Criteria for flagging unprocessed T1w data based on the MRIQC visual report

We illustrate in this section archetypal examples of T1w images we flagged. We could not illustrate data formatting issues on T1w images (criterion N) as none were spotted in this dataset.

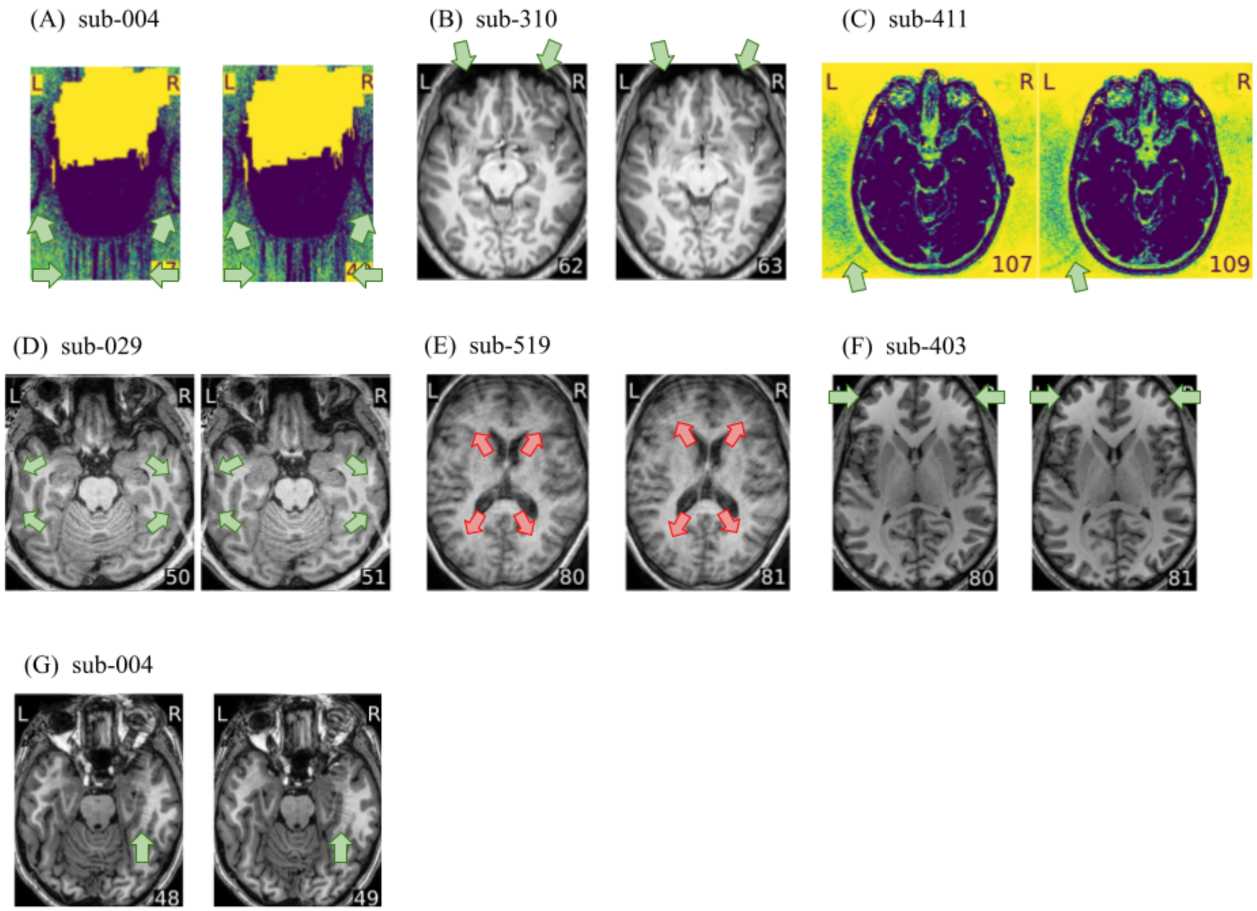

Figure S10. Flagging criteria for T1w based on the MRIQC visual report. (A) The background presents some structures that might contaminate the signals inside the brain (criterion J). **(B)** Susceptibility distortion of the anterior part of the prefrontal cortex is visible (criterion K). **(C)** The aliasing ghost is visible as a faint and shifted copy of the brain in the background (criterion L). **(D)** Because the FOV is too small, the part of the skull that was outside the FOV is reflected back on the contralateral temporal lobes, an artifact called wrap-around (criterion M). **(E)** Ripples caused by head motion blur the structure and destroy contrast (criterion O). **(F)** Intensity non-uniformity is visible as a slow and smooth drift in image intensity (criterion P). **(G)** Eye spillover is visible as a leakage of the signal from the eyes across the brain in the anterior-posterior phase-encoding direction (criterion Q).

1.3 Exclusion criteria of pre-processed data based on fMRIPrep visual report

We were unable to illustrate failure in normalization, brain delineation, surface reconstruction and co-registration as no subject exhibited those issues.

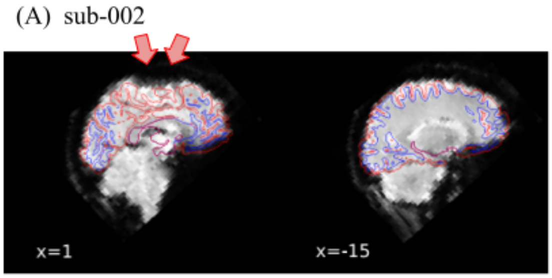

Figure S11. Residual susceptibility distortion. The observation of residual signal drop-out in the fMRI image led to the exclusion of the scan.

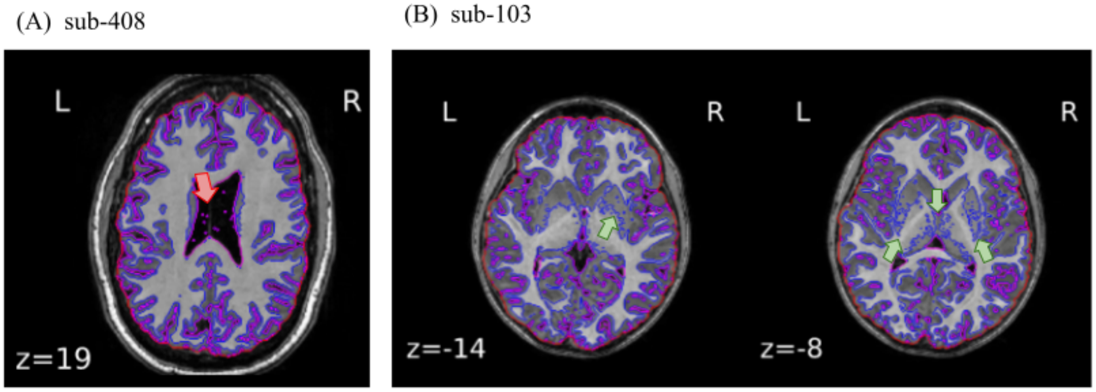

Figure S12. Error in brain tissue segmentation of T1w images. (A) The presence of noise compromises the segmentation leading to single voxels being excluded from the ventricle mask. The subject has thus been excluded from further analysis. **(B)** A series of spots are visible at the boundary between WM and GM. Those spots are due to partial volume effect and thus is a flaw of the fMRIPrep segmentation implementation not of the image quality.

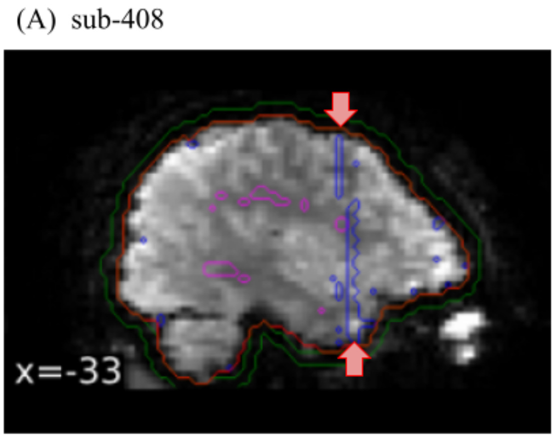

Figure S13. Regions identified for the extraction of nuisance regressors potentially cover neural signal sources. Because the tCompCor mask delineated in blue forms a straight line across the whole brain, it reflects artifactual patterns. This scan is hence excluded from further analysis.

2 Exclusion criteria occurrence

In this section, we give an overview on how often a scan was excluded based on each of the criteria. Given that most of the scans were excluded based on more than one criterion, the tables below report the exclusion criterion occurrence defined as the number of times the criterion was stated as a reason for exclusion.

Table S1. Occurrence of exclusion criteria for resting-state and task fMRI based on the MRIQC visual report. The criteria are sorted by total occurrence.

Exclusion criteria of unprocessed fMRI data based on MRIQC visual report	Exclusion criteria occurrence	Occurrence in resting-state fMRI data	Occurrence in task fMRI data
BA) Signal drop-out	121	113	8
C) Aliasing ghosts	63	58	5
EA) Structured crown region in the carpet plot due to motion peaks	36	25	11
D) Wrap-around that overlaps with the brain	26	6	20
A) Artifactual structures in the background	25	24	1
H) Vertical strikes in the sagittal plane of the standard deviation map	11	11	0
BB) Brain distortions	5	3	2
ED) Drift of unknown source	4	1	3
F) Artifacts detected with independent components analysis	4	0	4
EB) Structured crown region in the carpet plot due to periodic motion	3	3	0
I) Data formatting issues	3	2	1
G) Hyperintensity of single slices	2	2	0
EC) Structured crown region in the carpet plot due to coil failure	1	0	1

Table S2. Occurrence of T1w flagging criteria based on the MRIQC visual report. The criteria are sorted by total occurrence. One T1w image was exceptionally excluded based on the MRIQC visual report because of extreme motion-related ringing.

Flagging criteria of unprocessed T1w data based on MRIQC visual report	Flagging criteria occurrence	Occurrence in the resting-state subset	Occurrence in the task subset
O) Motion-related and Gibbs ringing	21	19 + 1 exclusion	1
J) Artifactual structures in the background	14	14	0
L) Aliasing ghost	9	9	0
KA) Signal drop-out	5	5	0
M) Wrap-around that overlaps with the brain	4	0	4
Q) Eye spillover	2	2	0
P) Extreme intensity non-uniformity	1	1	0
KB) Brain distortions	0	0	0
N) Data formatting issues	0	0	0

Table S3. Occurrence of exclusion criteria based on the fMRIPrep visual report. The criteria are sorted by total occurrence.

Exclusion criteria of unprocessed fMRI data based on MRIQC visual report	Exclusion criteria occurrence	Occurrence in resting-state subset	Occurrence in task subset
T) Residual susceptibility distortion	2	1	1
R) Failure in normalization to MNI space	0	0	0
S) Inaccurate brain mask	0	0	0
U) Error in brain tissue segmentation of T1w images	0	0	0
V) Surface reconstruction problem	0	0	0
W) Co-registration problem	0	0	0
X) Regions identified for the extraction of nuisance regressors potentially cover neural signal sources	0	0	0

3 Bash scripts to run the research topic dataset through MRIQC and fMRIPrep via docker

```
sub_nbr=($(seq -f "%03g" 1 1 30)) #subject numbers
bs=1 #batch size
for ((i=0; i<=${#sub_nbr[@]}; i+=bs)); do
    batch=${sub_nbr[@]:$i:1}
    echo ${batch[@]}
    docker run -u $( id -u ) -it --memory="8g" --rm -v
/data/datasets/QCResearchTopic/fmri-open-qc-task/:/data:ro -v
/data/derivatives/mriqc/v22.0.1/QCResearchTopic/fmri-open-qc-task/:/out -v
$HOME/tmp/mriqc/v22.0.1/QCResearchTopic/fmri-open-qc-task/:/work
nipreps/mriqc:22.0.1 /data /out --ica --verbose-report participant
--participant-label ${batch[@]} -w /work -vv
done
```

Listing S1. Execution of MRIQC with a Docker container on the task subset. MRIQC follows the standards laid out by BIDS-Apps (Gorgolewski et al. 2017). As such, the command line using containers is composed of a preamble configuring Docker, the name of the specific Docker image (nipreps/mriqc:22.0.1) and finally MRIQC's arguments. Because SynthStrip is a deep-learning based approach, the brain masking step requires at least 8GB of memory (specified by the --memory flag).

```
sub_nbr=(sub-101 sub-103 sub-710 sub-720) #subject numbers
bs=1 #batch size
for ((i=0; i<=${#sub_nbr[@]}; i+=bs)); do
   batch=${sub_nbr[@]:$i:1}
   echo ${batch[@]}
   docker run -u $( id -u ) -it --rm  --memory="8g" -v
$HOME/env/freesurfer_license.txt:/opt/freesurfer/license.txt:ro -v
/data/datasets/QCResearchTopic/fmri-open-qc-rest/:/data:ro -v
/data/derivatives/fmriprep/v22.0.0/QCResearchTopic/fmri-open-qc-rest/:/out -v
$HOME/tmp/fmriprep/v22.0.0/QCResearchTopic/fmri-open-qc-rest/:/work
nipreps/fmriprep:22.0.0 /data /out participant --participant-label ${batch[@]} -w
/work --nprocs 20 --omp-nthreads 16 -vv
done
```

Listing S2. Execution of fMRIPrep with a Docker container on the resting-state subset. Only the data that were not excluded based on the quality assessment of the MRIQC visual reports were run through fMRIPrep. fMRIPrep follows the standards laid out by BIDS-Apps (Gorgolewski et al. 2017). As such, the command line using containers is composed of a preamble configuring Docker, the name of the specific Docker image (nipreps/fmriprep:22.0.0) and finally fMRIPrep's arguments.

```
sub_nbr=(sub-002 sub-004 sub-014) #subject numbers
bs=1 #batch size
for ((i=0; i<=${#sub_nbr[@]}; i+=bs)); do
   batch=${sub_nbr[@]:$i:1}
   echo ${batch[@]}
   docker run -u $( id -u ) -it --rm  --memory="8g" -v
```

```
$HOME/env/freesurfer_license.txt:/opt/freesurfer/license.txt:ro -v
/data/datasets/QCResearchTopic/fmri-open-qc-task/:/data:ro -v
/data/derivatives/fmriprep/v22.0.0/QCResearchTopic/fmri-open-qc-task/:/out -v
$HOME/tmp/fmriprep/v22.0.0/QCResearchTopic/fmri-open-qc-task/:/work
nipreps/fmriprep:22.0.0 /data /out participant --participant-label ${batch[@]} -w
/work --nprocs 20 --omp-nthreads 16 -vv
done
```

Listing S3. Execution of fMRIPrep with a Docker container on the task subset. Only the data that were not excluded based on the quality assessment of the MRIQC visual reports were run through fMRIPrep. fMRIPrep follows the standards laid out by BIDS-Apps (Gorgolewski et al. 2017). As such, the command line using containers is composed of a preamble configuring Docker, the name of the specific Docker image (nipreps/fmriprep:22.0.0) and finally fMRIPrep's arguments.

4 Details of fMRIPrep preprocessing

Results included in this manuscript come from preprocessing performed using *fMRIPrep* 22.0.0 (Esteban, Markiewicz, et al. (2018); Esteban, Blair, et al. (2018); RRID:SCR_016216), which is based on *Nipype* 1.8.3 (K. Gorgolewski et al. (2011); K. J. Gorgolewski et al. (2018); RRID:SCR_002502).

4.1 Anatomical data preprocessing

A total of 1 T1-weighted (T1w) images were found within the input BIDS dataset.The T1-weighted (T1w) image was corrected for intensity non-uniformity (INU) with N4BiasFieldCorrection (Tustison et al. 2010), distributed with ANTs 2.3.3 (Avants et al. 2008, RRID:SCR_004757), and used as T1w-reference throughout the workflow. The T1w-reference was then skull-stripped with a *Nipype* implementation of the antsBrainExtraction.sh workflow (from ANTs), using OASIS30ANTs as target template. Brain tissue segmentation of cerebrospinal fluid (CSF), white-matter (WM) and gray-matter (GM) was performed on the brain-extracted T1w using fast (FSL 6.0.5.1:57b01774, RRID:SCR_002823, Zhang, Brady, and Smith 2001). Brain surfaces were reconstructed using recon-all (FreeSurfer 7.2.0, RRID:SCR_001847, Dale, Fischl, and Sereno 1999), and the brain mask estimated previously was refined with a custom variation of the method to reconcile ANTs-derived and FreeSurfer-derived segmentations of the cortical gray-matter of Mindboggle (RRID:SCR_002438, Klein et al. 2017). Volume-based spatial normalization to one standard space (MNI152NLin2009cAsym) was performed through nonlinear registration with antsRegistration (ANTs 2.3.3), using brain-extracted versions of both T1w reference and the T1w template. The following template was selected for spatial normalization: *ICBM 152 Nonlinear Asymmetrical template version 2009c* [Fonov et al. (2009), RRID:SCR_008796; TemplateFlow ID: MNI152NLin2009cAsym].

4.2 Functional data preprocessing

For each of the 1 BOLD runs found per subject (across all tasks and sessions), the following preprocessing was performed. First, a reference volume and its skull-stripped version were generated using a custom methodology of *fMRIPrep*. Head-motion parameters with respect to the BOLD reference (transformation matrices, and six corresponding rotation and translation parameters) are estimated before any spatiotemporal filtering using mcflirt (FSL 6.0.5.1:57b01774, Jenkinson et al.

2002). BOLD runs were slice-time corrected to 1.22s (0.5 of slice acquisition range 0s-2.45s) using 3dTshift from AFNI (Cox and Hyde 1997, RRID:SCR_005927). The BOLD time-series (including slice-timing correction when applied) were resampled onto their original, native space by applying the transforms to correct for head-motion. These resampled BOLD time-series will be referred to as *preprocessed BOLD in original space*, or just *preprocessed BOLD*. The BOLD reference was then co-registered to the T1w reference using bbregister (FreeSurfer) which implements boundary-based registration (Greve and Fischl 2009). Co-registration was configured with six degrees of freedom. Several confounding time-series were calculated based on the *preprocessed BOLD*: framewise displacement (FD), DVARS and three region-wise global signals. FD was computed using two formulations following Power (absolute sum of relative motions, Power et al. (2014)) and Jenkinson (relative root mean square displacement between affines, Jenkinson et al. (2002)). FD and DVARS are calculated for each functional run, both using their implementations in *Nipype* (following the definitions by Power et al. 2014). The three global signals are extracted within the CSF, the WM, and the whole-brain masks. Additionally, a set of physiological regressors were extracted to allow for component-based noise correction (*CompCor*, Behzadi et al. 2007). Principal components are estimated after high-pass filtering the *preprocessed BOLD* time-series (using a discrete cosine filter with 128s cut-off) for the two *CompCor* variants: temporal (tCompCor) and anatomical (aCompCor). tCompCor components are then calculated from the top 2% variable voxels within the brain mask. For aCompCor, three probabilistic masks (CSF, WM and combined CSF+WM) are generated in anatomical space. The implementation differs from that of Behzadi et al. in that instead of eroding the masks by 2 pixels on BOLD space, a mask of pixels that likely contain a volume fraction of GM is subtracted from the aCompCor masks. This mask is obtained by dilating a GM mask extracted from the FreeSurfer's *aseg* segmentation, and it ensures components are not extracted from voxels containing a minimal fraction of GM. Finally, these masks are resampled into BOLD space and binarized by thresholding at 0.99 (as in the original implementation). Components are also calculated separately within the WM and CSF masks. For each CompCor decomposition, the *k* components with the largest singular values are retained, such that the retained components' time series are sufficient to explain 50 percent of variance across the nuisance mask (CSF, WM, combined, or temporal). The remaining components are dropped from consideration. The head-motion estimates calculated in the correction step were also placed within the corresponding confounds file. The confound time series derived from head motion estimates and global signals were expanded with the inclusion of temporal derivatives and quadratic terms for each (Satterthwaite et al. 2013). Frames that exceeded a threshold of 0.5 mm FD or 1.5 standardized DVARS were annotated as motion outliers. Additional nuisance timeseries are calculated by means of principal components analysis of the signal found within a thin band (*crown*) of voxels around the edge of the brain, as proposed by (Patriat, Reynolds, and Birn 2017). The BOLD time-series were resampled into standard space, generating a *preprocessed BOLD run in MNI152NLin2009cAsym space*. First, a reference volume and its skull-stripped version were generated using a custom methodology of *fMRIPrep*. All resamplings can be performed with *a single interpolation step* by composing all the pertinent transformations (i.e. head-motion transform matrices, susceptibility distortion correction when available, and co-registrations to anatomical and output spaces). Gridded (volumetric) resamplings were performed using antsApplyTransforms (ANTs), configured with Lanczos interpolation to minimize the smoothing effects of other kernels (Lanczos 1964). Non-gridded (surface) resamplings were performed using mri_vol2surf (FreeSurfer).

Many internal operations of *fMRIPrep* use *Nilearn* 0.9.1 (Abraham et al. 2014, RRID:SCR_001362), mostly within the functional processing workflow. For more details of the pipeline, see the section corresponding to workflows in *fMRIPrep*'s documentation.

4.3 References

Abraham, Alexandre, Fabian Pedregosa, Michael Eickenberg, Philippe Gervais, Andreas Mueller, Jean Kossaifi, Alexandre Gramfort, Bertrand Thirion, and Gael Varoquaux. 2014. "Machine Learning for Neuroimaging with Scikit-Learn." *Frontiers in Neuroinformatics* 8. https://doi.org/10.3389/fninf.2014.00014.

Avants, B. B., C. L. Epstein, M. Grossman, and J. C. Gee. 2008. "Symmetric Diffeomorphic Image Registration with Cross-Correlation: Evaluating Automated Labeling of Elderly and Neurodegenerative Brain." *Medical Image Analysis* 12 (1): 26–41. https://doi.org/10.1016/j.media.2007.06.004.

Behzadi, Yashar, Khaled Restom, Joy Liau, and Thomas T. Liu. 2007. "A Component Based Noise Correction Method (CompCor) for BOLD and Perfusion Based fMRI." *NeuroImage* 37 (1): 90–101. https://doi.org/10.1016/j.neuroimage.2007.04.042.

Cox, Robert W., and James S. Hyde. 1997. "Software Tools for Analysis and Visualization of fMRI Data." *NMR in Biomedicine* 10 (4-5): 171–78. https://doi.org/10.1002/(SICI)1099-1492(199706/08)10:4/5<171::AID-NBM453>3.0.CO;2-L.

Dale, Anders M., Bruce Fischl, and Martin I. Sereno. 1999. "Cortical Surface-Based Analysis: I. Segmentation and Surface Reconstruction." *NeuroImage* 9 (2): 179–94. https://doi.org/10.1006/nimg.1998.0395.

Esteban, Oscar, Ross Blair, Christopher J. Markiewicz, Shoshana L. Berleant, Craig Moodie, Feilong Ma, Ayse Ilkay Isik, et al. 2018. "fMRIPrep 22.0.0." *Software*. https://doi.org/10.5281/zenodo.852659.

Esteban, Oscar, Christopher Markiewicz, Ross W Blair, Craig Moodie, Ayse Ilkay Isik, Asier Erramuzpe Aliaga, James Kent, et al. 2018. "fMRIPrep: A Robust Preprocessing Pipeline for Functional MRI." *Nature Methods*. https://doi.org/10.1038/s41592-018-0235-4.

Fonov, VS, AC Evans, RC McKinstry, CR Almli, and DL Collins. 2009. "Unbiased Nonlinear Average Age-Appropriate Brain Templates from Birth to Adulthood." *NeuroImage* 47, Supplement 1: S102. https://doi.org/10.1016/S1053-8119(09)70884-5.

Gorgolewski, K., C. D. Burns, C. Madison, D. Clark, Y. O. Halchenko, M. L. Waskom, and S. Ghosh. 2011. "Nipype: A Flexible, Lightweight and Extensible Neuroimaging Data Processing Framework in Python." *Frontiers in Neuroinformatics* 5: 13. https://doi.org/10.3389/fninf.2011.00013.

Gorgolewski, Krzysztof J., Oscar Esteban, Christopher J. Markiewicz, Erik Ziegler, David Gage Ellis, Michael Philipp Notter, Dorota Jarecka, et al. 2018. "Nipype." *Software*. https://doi.org/10.5281/zenodo.596855.

Greve, Douglas N, and Bruce Fischl. 2009. "Accurate and Robust Brain Image Alignment Using Boundary-Based Registration." *NeuroImage* 48 (1): 63–72. https://doi.org/10.1016/j.neuroimage.2009.06.060.

Jenkinson, Mark, Peter Bannister, Michael Brady, and Stephen Smith. 2002. “Improved Optimization for the Robust and Accurate Linear Registration and Motion Correction of Brain Images.” *NeuroImage* 17 (2): 825–41. https://doi.org/10.1006/nimg.2002.1132.

Klein, Arno, Satrajit S. Ghosh, Forrest S. Bao, Joachim Giard, Yrjö Häme, Eliezer Stavsky, Noah Lee, et al. 2017. “Mindboggling Morphometry of Human Brains.” *PLOS Computational Biology* 13 (2): e1005350. https://doi.org/10.1371/journal.pcbi.1005350.

Lanczos, C. 1964. “Evaluation of Noisy Data.” *Journal of the Society for Industrial and Applied Mathematics Series B Numerical Analysis* 1 (1): 76–85. https://doi.org/10.1137/0701007.

Patriat, Rémi, Richard C. Reynolds, and Rasmus M. Birn. 2017. “An Improved Model of Motion-Related Signal Changes in fMRI.” *NeuroImage* 144, Part A (January): 74–82. https://doi.org/10.1016/j.neuroimage.2016.08.051.

Power, Jonathan D., Anish Mitra, Timothy O. Laumann, Abraham Z. Snyder, Bradley L. Schlaggar, and Steven E. Petersen. 2014. “Methods to Detect, Characterize, and Remove Motion Artifact in Resting State fMRI.” *NeuroImage* 84 (Supplement C): 320–41. https://doi.org/10.1016/j.neuroimage.2013.08.048.

Satterthwaite, Theodore D., Mark A. Elliott, Raphael T. Gerraty, Kosha Ruparel, James Loughead, Monica E. Calkins, Simon B. Eickhoff, et al. 2013. “An improved framework for confound regression and filtering for control of motion artifact in the preprocessing of resting-state functional connectivity data.” *NeuroImage* 64 (1): 240–56. https://doi.org/10.1016/j.neuroimage.2012.08.052.

Tustison, N. J., B. B. Avants, P. A. Cook, Y. Zheng, A. Egan, P. A. Yushkevich, and J. C. Gee. 2010. “N4itk: Improved N3 Bias Correction.” *IEEE Transactions on Medical Imaging* 29 (6): 1310–20. https://doi.org/10.1109/TMI.2010.2046908.

Zhang, Y., M. Brady, and S. Smith. 2001. “Segmentation of Brain MR Images Through a Hidden Markov Random Field Model and the Expectation-Maximization Algorithm.” *IEEE Transactions on Medical Imaging* 20 (1): 45–57. https://doi.org/10.1109/42.906424.
